# Deleterious Effect of the IL-23/IL-17A Axis and γδT Cells on Left Ventricular Remodeling After Myocardial Infarction

**DOI:** 10.1161/JAHA.112.004408

**Published:** 2012-10-25

**Authors:** Xiaoxiang Yan, Takashi Shichita, Yoshinori Katsumata, Tomohiro Matsuhashi, Hideyuki Ito, Kentaro Ito, Atsushi Anzai, Jin Endo, Yuichi Tamura, Kensuke Kimura, Jun Fujita, Ken Shinmura, Weifeng Shen, Akihiko Yoshimura, Keiichi Fukuda, Motoaki Sano

**Affiliations:** Department of Cardiology, Keio University School of Medicine, Tokyo, Japan (X.Y., Y.K., T.M., H.I., K.I., A.A., J.E., Y.T., K.K., J.F., K.F., M.S.); Department of Microbiology and Immunology, Keio University School of Medicine, Tokyo, Japan (T.S., A.Y.); Department of Geriatric Medicine, Keio University School of Medicine, Tokyo, Japan (K.S.); Department of Cardiology, Ruijin Hospital, Shanghai Jiaotong University School of Medicine, Shanghai, China (W.S.)

**Keywords:** heart failure, immune system, inflammation, myocardial infarction, remodeling

## Abstract

**Background:**

Left ventricular (LV) remodeling leads to chronic heart failure and is a main determinant of morbidity and mortality after myocardial infarction (MI). At the present time, therapeutic options to prevent LV remodeling are limited.

**Methods and Results:**

We created a large MI by permanent ligation of the coronary artery and identified a potential link between the interleukin (IL)–23/IL-17A axis and γδT cells that affects late-stage LV remodeling after MI. Despite the finsinf that infarct size 24 hours after surgery was similar to that in wild-type mice, a deficiency in IL-23, IL-17A, or γδT cells improved survival after 7 days, limiting infarct expansion and fibrosis in noninfarcted myocardium and alleviating LV dilatation and systolic dysfunction on day 28 post-MI. M_1_ macrophages and neutrophils were the major cellular source of IL-23, whereas >90% of IL-17A-producing T cells in infarcted heart were CD4^−^ TCRγδ^+^ (γδT) cells. Toll-like receptor signaling and IL-1β worked in concert with IL-23 to drive expansion and IL-17A production in cardiac γδT cells, whereas the sphingosine-1-phosphate receptor and CCL20/CCR6 signaling pathways mediated γδT cell recruitment into infarcted heart. IL-17A was not involved in the acute inflammatory response, but it functioned specifically in the late remodeling stages by promoting sustained infiltration of neutrophils and macrophages, stimulating macrophages to produce proinflammatory cytokines, aggravating cardiomyocyte death, and enhancing fibroblast proliferation and profibrotic gene expression.

**Conclusions:**

The IL-23/IL-17A immune axis and γδT cells are potentially promising therapeutic targets after MI to prevent progression to end-stage dilated cardiomyopathy.

## Introduction

Left ventricular (LV) remodeling leads to chronic heart failure and is a main determinant of morbidity and mortality after myocardial infarction (MI).^1^ Myocardial infarct volume and subsequent adverse LV remodeling (dilation and fibrosis) determine the degree of LV dysfunction^2^; however, current therapeutic options to prevent LV remodeling are limited. Timely revascularization of ischemic myocardium followed by standard therapy with renin–angiotensin–aldosterone inhibitors and β-blockers can alleviate post-MI remodeling.^2–5^ However, the last decade has witnessed a paradoxical increase in the incidence of heart failure within 30 days post-MI.^6^ More therefore needs to be done to prevent progressive LV dysfunction during hospitalization following MI.

Activation of the immune system is critically involved in adverse LV remodeling after MI.^7–10^ The immune response after tissue damage is primarily responsible for wound healing, although this response can also exacerbate tissue injury if the inflammatory cascades are inappropriately or excessively activated.^9–11^ Attempts at introducing nonselective anti-inflammatory glucocorticoids for the treatment of acute MI produced conflicting results.^12^ There is a concern that impairment of the wound healing process by glucocorticoids may facilitate wall thinning and ventricular rupture.^12^ To develop successful immunomodulatory therapies, we need to delineate a distinct subset of cells and cytokines that have only minor effects on the acute wound healing process but that are strongly involved in the smoldering inflammation responsible for adverse LV geometric and functional remodeling.

Interleukin (IL)-23, comprising an IL-12/IL-23p40 subunit and a p19 subunit, interacts with its receptor IL-23R (composed of IL-23R and IL12R-β1) to stimulate IL-17A production, in which the key transcription factor RORγt regulates both IL-17A and IL-23R expression.^13–15^ IL-23- and IL-17A-producing cells are involved in the pathogenesis of various inflammatory diseases such as atherosclerosis,^16^ allergies,^17^ autoimmune diseases,^14,15,18–20^ and allograft transplantation.^21^ Recent reports also implicated IL-17A has having a key role in cardiac ischemia–reperfusion injury^22,23^ and postmyocarditis LV remodeling,^19^ prompting us to examine the impact of these cytokines on post-MI cardiac remodeling.

## Methods

### Mice

IL-17A-knockout (KO)^24^ and IL-23p19-KO (IL-23-KO)^20^ mice were described previously. TCRγδ-KO mice were purchased from Jackson Laboratories. The TLR2-KO, TLR4-KO, and TLR2/4 double-knockout mice were a generous gift from Dr Shizuo Akira (Osaka University) and described previously.^25^ All mice were bred on the C57BL/6 background, and 10- to 16-week-old male mice were used in this study. All animal experiments were reviewed and approved by the Institutional Animal Care and Use Committee at the Keio University School of Medicine.

### Induction of MI and Infarct Size Evaluation

Mice were subjected to a permanent (MI) ligation of the left anterior descending artery or to a sham operation without ligation as described previously.^26^ In brief, mice were lightly anesthetized with diethyl ether, intubated, and then fully anesthetized with 1.0% to 1.5% isoflurane gas while being mechanically ventilated with a rodent respirator. The chest cavity was opened via left thoracotomy to expose the heart such that the left anterior descending coronary could be visualized by microscopy and permanently ligated with a 7-0 silk suture at the site of its emergence from the left atrium. Complete occlusion of the vessel was confirmed by the presence of myocardial blanching in the perfusion bed. Mice that died during recovery from anesthesia were excluded from the analysis. Sham-operated animals underwent the same procedure without coronary artery ligation. In the functional experiments, the sham operation was done and waited for 28 days; however, in the immune cells infiltration experiments and qPCR experiments, the sham operation was done and sacrifice was on day 2 or day 7. In addition, we separately verified that the numbers of macrophages, T cells, and neutrophils in the heart remained constant on days 1, 4, 7, and 14 after the sham operation. To evaluate the infarct size on day 1 after MI, hearts were weighed and frozen at −80°C. The frozen hearts were cut transversely into 1-mm-thick slices using a Mouse Heart Slicer Matrix and stained with 2% triphenyltetrazolium chloride (TTC) in PBS (pH 7.4) for 20 minutes in a 37°C water bath. After fixation for 4 to 6 hours in 10% neutral buffered formaldehyde, both sides of each slice were photographed. Viable myocardium stained brick red, and infarct tissues appeared pale white. Infarct and LV area were measured by automated planimetry using Image J software (version 1.43u, National Institutes of Health), with the infarct size expressed as a percentage of the total LV area.

### Cell Preparation for Flow Cytometry

At each time, mice were deeply anesthetized and intracardially perfused with 40 mL of ice-cold PBS to exclude blood cells. The heart was dissected, minced with fine scissors, and then enzymatically digested with a cocktail of type II collagenase (Worthington Biochemical Corporation, Lakewood, NJ), elastase (Worthington Biochemical Corporation), and *DNase*I (Sigma, St. Louis, MO) for 1.5 hours at 37°C with gentle agitation. After digestion, the tissue was triturated and passed through a 70-μm cell strainer. Leukocyte-enriched fractions were isolated by a 37% to 70% Percoll (GE Healthcare) density gradient centrifugation as described elsewhere.^27^ Cells were removed from the interface and washed with RPMI-1640 cell culture medium for further analysis. Spleens were removed, homogenized, and then passed through a 70-μm nylon mesh in PBS. After the addition of red blood cell lysis buffer (eBioscience) to exclude erythrocytes, the single-cell suspension in PBS was refiltered through a 70-μm nylon mesh to remove connective tissue.

### Flow Cytometric Analysis

Cell suspensions isolated from spleen and leukocyte-enriched fractions from heart were analyzed by flow cytometry. To block nonspecific binding of antibodies to Fcγ receptors, isolated cells were first incubated with anti-CD16/32 antibody (2.4G2; BD Biosciences) at 4°C for 5 minutes. Subsequently, the cells were stained with a mixture of antibodies at 4°C for 20 minutes. Results were expressed as cell number per heart. Flow-cytometric analysis and sorting were performed on a FACSAria instrument (BD Biosciences) and analyzed using FlowJo software (Tree Star).

### Antibodies Used for Flow Cytometry

Anti-CD45-FITC (30F11.1; eBioscience), anti-CD45-PE (30-F11; BD Biosciences), anti-CD11b-PerCP-Cy5.5, anti-CD11b FITC (M1/70; eBioscience), anti-CD11b-PE (M1/70; BD Biosciences), anti-CD3e-FITC, anti-CD3e-APC (145-2C11; eBioscience), anti-CD3e-PE (145-2C11; BD Biosciences), anti-CD19-PE (1D3; BD Biosciences), anti-CD4-PE (GK1.5; eBioscience), anti-CD8a-PE (53-6.7; eBioscience), anti-TCR-γδ-PE (GL3; Biolegend), anti-NK1.1-PE (PK136; eBioscience), anti-CD11c-APC, anti-CD11c-PE (N418; Biolegend), anti-MHC-II (I-A/I-E)-PE (M5/114.15.2; eBioscience), anti-Gr-1-APC, anti-Gr-1-Alexa Fluor488 (RB6-8C5; eBioscience), anti-F4/80-FITC, anti-F4/80-PE (BM8; Biolegend), anti-F4/80-APC (BM8; eBioscience), anti-Ly-6G-PE (1A8; BD Biosciences), anti-CD206 (MMR)-AlexaFluor647 (MR5D3; Biolegend), anti-CCR6-Alexa Fluor(R) 647 (140706; BD Biosciences), anti-CXCR2-APC (FAB2164A; RD system), anti-Thy1.2-APC (53-2.1; eBioscience), anti-CD31-PerCP-eFluor710 (390; eBioscience), anti-IL-17A-APC (eBio17B7; eBioscience), and anti-IFN-γ-APC (XMG1.2; eBioscience) antibodies were used for flow-cytometric analysis in this study.

### Intracellular Cytokine Staining

For surface and intracellular cytokine staining, single cells prepared from spleen and heart were restimulated for 4.5 hours with 50 ng/mL phorbol 12-myristate 13-acetate (PMA; Sigma-Aldrich) and 1 μg/mL ionomycin (Sigma-Aldrich) in the presence of Golgistop (Cytofix/Cytoperm Plus Kit with Golgistop, BD Biosciences). Surface staining was performed for 20 minutes with the corresponding mixture of fluorescently labeled antibodies. After fixation and permeabilization, the cells were incubated for 30 minutes at 4°C with anti-IL-17A-APC and anti-IFN-γ-APC (eBioscience).

### In Vitro Cardiac Cell Stimulation

Mouse heart cells were prepared from infarcted heart, and CD45 MicroBeads were used to enrich for heart-derived leukocytes. Cells were stimulated with 10 ng/mL rmIL-23, 10 ng/mL rmIL-1β (R&D Systems), 100 ng/mL LPS (Sigma-Aldrich), and 1 μg/mL Pam3CSK4 (InvivoGen) for 3 days in the presence or absence of 3 μg/mL IL-1RI (IL-1 receptor I) antibody. The supernatants were harvested and assayed for IL-17A levels by ELISA (R&D Systems). After 3 days stimulation, cells were also restimulated for 4.5 hours with PMA and ionomycin in the presence of Golgistop for intracellular IL-17A staining. For the γδT cell proliferation assay, cardiac cells were stained with 5(6)-carboxyfluorescein diacetate *N*-succinimidyl ester (CFSE; Sigma-Aldrich) according to the manufacturer's recommendations. Briefly, cells were stained in 10 μmol/L CFSE in PBS at 37°C for 5 minutes and then washed 3 times with cold PBS. Labeled cells were stimulated for 3 days, and then stained for surface markers CD3 and TCRγδ prior to flow cytometry.

### Isolation of Neonatal and Adult Cardiomyocytes and Nonmyocytes

Neonatal ventricles from 1-day-old C57BL6/J mice were minced and digested with collagenase type II (Worthington) solution.^28^ To enrich for cardiomyocytes, the cells were preplated for 2 hours to remove nonmyocytes. The unattached viable cells, which were rich in cardiomyocytes, were plated on gelatin-coated plastic dishes and treated with Ara C (Sigma) to inhibit nonmyocyte proliferation. Using this protocol, we consistently obtained cell populations containing ≥90% to 95% cardiomyocytes. Nonmyocyte cells that attached to the dishes were cultured in DMEM supplemented with 10% FBS and allowed to grow to confluence; these were then trypsinized and passaged at 1 in 4. This procedure yielded cell cultures that were almost exclusively fibroblasts by the first passage. Experiments were carried out after 2 passages.

Adult cardiomyocytes were isolated using the Langendorff perfusion method as previously described.^28^ For sorting of fibroblasts, macrophages, endothelial cells, and lymphocytes, single cells prepared from infarcted heart were incubated with APC-conjugated anti-Thy1 antibody (eBioscience), PerCP-eFluor710-conjugated anti-CD31 antibody (eBioscience), FITC-conjugated anti-CD45 antibody (eBioscience), and PE-conjugated anti-F4/80 antibody (eBioscience), after which they were analyzed and sorted using a FACSAria (BD Biosciences) and FlowJo software.

### Quantitative Real-Time PCR

Total RNA samples from sorted cells, cultured cells, and heart tissue were prepared using using an RNeasy Mini Kit (Qiagen) or Trizol reagent (Invitrogen), according to the manufacturer's instructions. A First-strand cDNA synthesis kit (Invitrogen) was used for cDNA synthesis. Quantitative real-time PCR was performed using the ABI Prism 7700 sequence detection system (Applied Biosystems). Predesigned gene-specific primer and probe sets (Taqman Gene Expression Assays, Applied Biosystems) were used. The 18S ribosomal RNA was amplified as an internal control.

### Measurement of Cytokines by ELISA

LVs were homogenized in PBS containing protease inhibitors (Sigma-Aldrich). Supernatants were collected after centrifugation and stored at −80°C. The concentrations of IL-23, IL-1β, and IL-17A in LV lysates and culture supernatants were measured by Quantikine ELISA kits (R&D Systems).

### Gelatin Zymography

To evaluate the activity of gelatinase, matrix metalloproteinase 9 (MMP9), and MMP2, gelatin zymography was performed. Equal volumes containing 35 μg of protein were loaded into each lane of 10% gelatin zymogram gels (Novex, Invitrogen). After running at 125 V for 90 minutes, the gels were incubated in zymogram renaturing buffer for 30 minutes at room temperature with gentle agitation, equilibrated with zymogram developing buffer for 30 minutes, and then further incubated in zymogram developing buffer at 37°C overnight with gentle agitation. After washing with deionized water, the gels were stained with Coomassie blue for 90 minutes followed by destaining with deionized water. The presence of different MMPs was identified on the basis of their molecular weight. The gels were photographed using MF-ChemiBIS (DNR Bio-Imaging Systems) and analyzed using Image J software (version 1.43u, National Institutes of Health).

### Low Serum Hypoxia and Reoxygenation

An anaerobic jar containing an Anaero Pack (Mitsubishi Gas Chemical) was used to expose the cells to hypoxic stress.^29^ Cultured cardiomyocytes were serum starved in DMEM with 0.5% FBS, and then exposed to hypoxic stress and/or IL-17A stimulation (421-ML, R&D systems). Neutralizing anti-IL-17A antibody (AF-421-NA) was administered 2 hours before hypoxic stress. After 12 hours of exposure to hypoxia, the medium was replaced with 10% FBS-containing DMEM (reoxygenation medium). Cell viability was determined by a LIVE/DEAD Viability/Cytotoxicity Assay Kit (Invitrogen) on the basis of the simultaneous determination of live and dead cells with the calcein AM and ethidium homodimer-1 probes, which are specific for intracellular esterase activity and membrane integrity, respectively. The cells were imaged with a fluorescence microscope (BZ-9000; Keyence): live cells were labeled green, whereas nuclei of dead cells were labeled red.

### CCK-8 Assay

Mouse fibroblasts were cultivated in DMEM supplemented with 10% fetal bovine serum (FBS), penicillin, and streptomycin in a cell incubator with 5% CO_2_ at 37°C. Cell proliferation was analyzed by the CCK-8 assay (Dojindo Molecular Technologies, Japan) as directed by the manufacturer. Fibroblasts were seeded at 4000 cells per well in 96-well culture plates and cultured for 72 hours for cell proliferation analysis.

### Morphometric Analysis

Heart tissue was fixed in formalin, embedded in paraffin, and cut into 5-μm-thick sections. Hematoxylin and eosin (H&E) and Azan staining were performed on paraffin-embedded sections to determine the morphological effects, infarct size, and extent of cardiac fibrosis. The infarct size was calculated as total infarct circumference divided by total LV circumference×100, as described previously.^26^ In addition, for each Azan-stained section, 20 microscopic fields (×400 magnification) were randomly chosen (BZ-9000; Keyence, Osaka, Japan), and the area of myocardial fibrosis in noninfarct and infarct areas was measured and analyzed using analysis software (BZ image analyzer II; Keyence).

### Echocardiography

Transthoracic echocardiography was performed with a Vevo 2100 instrument (VisualSonics) equipped with an MS-400 imaging transducer. Mice were kept awake without anesthesia during the echocardiographic examination to minimize data deviation, and heart rate was maintained at ≍550 to 650 bpm in all mice. M-mode tracings were recorded through the anterior and posterior LV walls at the papillary muscle level to measure LV end-diastolic dimension (LVEDD) and LV end-systolic dimension (LVESD). LV fractional shortening (FS) was calculated according to the following formula: LV FS=([LVEDD−LVESD]/LVEDD)×100.

### Hemodynamics

Cardiac catheterization studies were performed using a 1.4 French microtip catheter (SPR-671, Millar Instruments, Houston, TX) under sedation using 1.5% isoflurane inhalation with spontaneous respiration. LV end-systolic pressure (LVESP), maximum rate of isovolumic pressure development, and minimum rate of isovolumic pressure decay were measured using analysis software (PowerLab, AD Instruments). Ten sequential beats were averaged for each measurement.

### Immunohistochemistry

For immunostaining of γδT lymphocytes in the murine ischemic heart tissue, we used frozen sections as described elsewhere.^27^ Cryostat sections (6 μm) were air-dried and then fixed in cold acetone at room temperature for 10 minutes. Endogenous peroxidase activity was blocked with 0.3% hydrogen peroxide (Sigma-Aldrich) in PBS for 20 minutes. Blocking with normal goat serum was applied for 1 hour at room temperature. Sections were washed with PBS and then incubated overnight at 4°C with hamster-anti-mouse TCRγδ antibody (5 μg/mL; clone GL-3, BD Biosciences/Pharmingen). After 3 washes with PBS, the sections were incubated with HRP-conjugated secondary antibody (mouse anti-hamster IgG-HRP, Santa Cruz Biotechnology) at room temperature for 4 hours in the dark. After washing with PBS, staining was developed with 3, 3′-diaminobenzidine tetrahydrochloride (Histofine DAB Substrate Kit, Nichirei). To visualize the cardiomyocytes, a sequential section was stained with anti-α-actinin antibody (Sigma-Aldrich).

### Apoptosis Analysis

Frozen sections of the heart samples fixed by 4% paraformaldehyde were subjected to TUNEL staining using a commercially available kit (In Situ Apoptosis Detection Kit; Takara Biomedicals) as directed by the manufacturer. Anti-α-actinin (Sigma-Aldrich) was used to identify cardiomyocytes. TUNEL-positive nuclei were counted, and the data were normalized per total nuclei identified by DAPI staining (Invitrogen) in the same sections.

### FTY720 Administration

FTY720 was dissolved at 5 mg/mL in DMSO, and then diluted with distilled water. FTY720 (1 mg/kg body weight) was administered intravenously from day 0 to day 5 post-MI, and leukocyte infiltration into the heart was analyzed by flow cytometry on day 6 post-MI.

### Statistics

Values are presented as mean±SEM. Comparisons between groups were made using a Mann–Whitney *U* test, whereas data among multiple groups were compared using either the Kruskall–Wallis test with Dunn's multiple comparisons test or 2-way ANOVA followed by Tukey's post hoc analysis, as appropriate. Survival distributions were estimated by the Kaplan–Meier method and compared by the log-rank test. A value of *P*<0.05 was considered statistically significant. Statistical analysis was performed with GraphPad Prism 5.0 (Graph Pad Prism Software Inc, San Diego, CA) and SPSS 15.0 for Windows (SPSS, Inc, Chicago, IL).

## Results

### IL-23 and IL-17A Adversely Affected Post-MI Cardiac Remodeling

**Figure 1.**
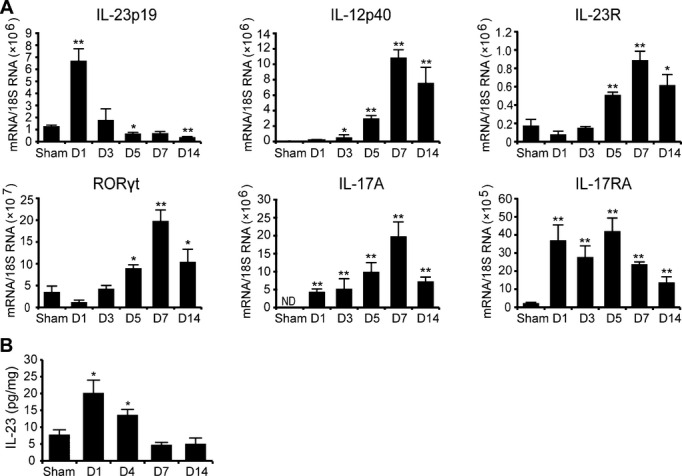
Quantification of temporal dynamics of IL-23/IL-17A axis in the infarcted heart. A, Time course of changes in mRNA expression of IL-23p19, IL-12p40, IL-23 receptor (IL-23R), RORγt, IL-17A, and IL-17 receptor A (IL-17RA) in heart tissue after MI. The levels of each transcript were normalized to 18S (n=4 to 6 each). *P<0.05, **P<0.01 vs sham. B, IL-23 protein levels were measured by ELISA in left ventricular tissues after MI. Values were normalized to total protein concentration in left ventricular tissues (n=4 each). *P<0.05 vs sham. Data in (A) and (B) were analyzed by Kruskall–Wallis tests with Dunn's multiple comparisons.

We first investigated the dynamics of IL-23 and IL-17A in post-MI heart. Expression of IL-23p19 subunit mRNA increased rapidly in infarcted heart on day 1 post-MI, returning to near-baseline levels by 3 days post-MI (Figure 1A). In contrast, expression of IL-23 receptor (IL-23R), IL-12/IL-23p40 subunit, RORγt, and IL-17A mRNA increased more gradually, peaking 7 days post-MI. Expression of IL-17 receptor A (IL-17RA) mRNA increased on day 1 post-MI and remained elevated until 14 days post-MI. ELISA analysis showed that the levels of IL-23 protein in post-MI heart increased rapidly to a peak at 24 hours, and then remained substantially elevated until at least day 4 post-MI (Figure 1B).

**Figure 2.**
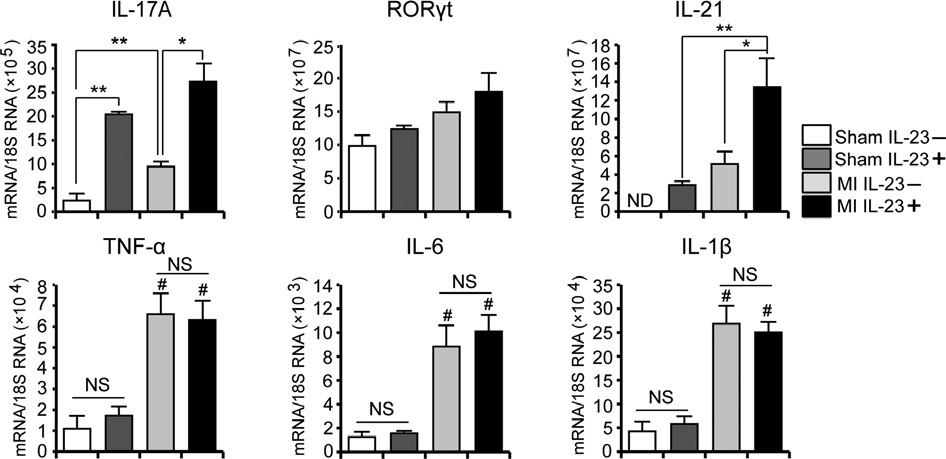
There were leukocytes that could produce IL-17A in response to IL-23 in the heart. The enriched leukocytes collected from either sham-operated or infarcted heart (on day 6 post-MI) were stimulated with IL-23 (10 ng/mL) for 24 hours and then analyzed for gene expressions, as indicated by quantitative RT-PCR (n=4). NS, not significant; *P<0.05, **P<0.01, #*P*<0.05 vs corresponding sham group. Data were analyzed by 2-way ANOVA followed by Tukey's post hoc analysis.

We next investigated whether IL-23 acts as an upstream regulator of IL-17A in infarcted heart. The leukocyte-enriched fraction was collected from sham-operated and infarcted hearts on day 6 post-MI, and cells were cultured with either vehicle or IL-23 (10 ng/mL) for 24 hours (Figure 2). Expression of IL-17A and IL-21 mRNA was markedly increased by IL-23 treatment in both leukocyte-enriched fractions, whereas expression of tumor necrosis factor (TNF)-α, IL-6, and IL-1β mRNA was unaffected. IL-23 also did not affect RORγt expression, indicating that RORγt is constitutively expressed in a subset of leukocytes.

**Figure 3.**
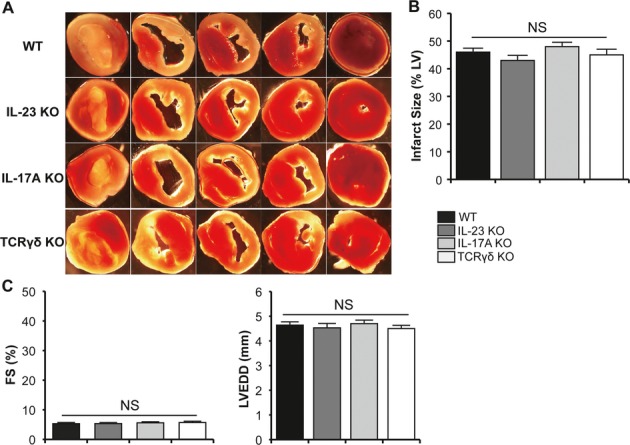
Infarct size and cardiac function were comparable among different mice. A, Twenty-four hours after MI, hearts were removed and stained with 2,3,5-triphenyltetrazolium chloride (TTC) for measurement of infarct area. Viable parts of the heart appear red and the infarct area white. B, Quantification of the infarct area shows a comparable infarct size among WT, IL-23-KO, IL-17A-KO, and TCRγδ-KO mice on day 1 after MI (n=5). C, There was no significant difference in left ventricular fractional shortening (FS) or left ventricular end diastolic diameter (LVEDD) on day 1 after MI, as evaluated by echocardiography (n=5). WT indicates wild-type; KO, knockout; LV, left ventricular; NS, not significant; and MI, myocardial infarction. Statistical analysis was performed by Kruskall–Wallis tests (B and C).

We then examined the functional significance of the IL-23/IL-17A axis in post-MI cardiac remodeling. On day 1 post-MI, infarct size (determined by TTC staining) and FS and LVEDD (assessed by echocardiography) in IL-17A-KO and IL-23-KO mice were comparable to those of wild-type (WT) mice (Figure 3A through 3C).

**Figure 4.**
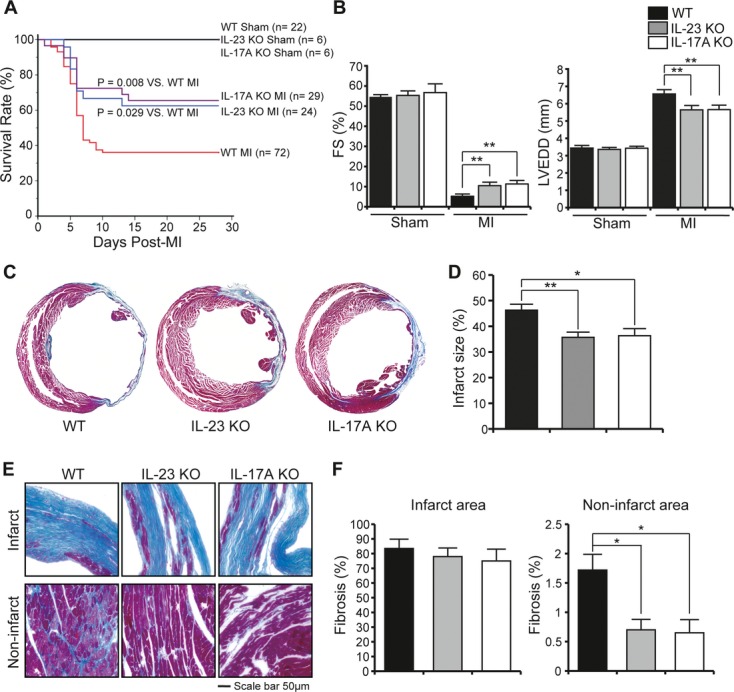
Deficiency of IL-23 and IL-17A conferred resistance to LV remodeling on day 28 post-MI. A, Kaplan–Meier survival analysis in WT, IL-23-KO, and IL-17A-KO mice after MI or sham operation. B, Echocardiographic analysis of fractional shortening (FS) and left ventricular end diastolic diameter (LVEDD) after MI or sham operation (n=9 to 16). C, Azan staining of cardiac sections in WT, IL-23-KO, and IL-17A-KO mice after MI. D, Infarct size determined with Azan staining of sections (n=9 to 16). E, Representative Azan-stained images of infarcted and noninfarcted areas 28 days after MI. Blue staining indicates fibrosis. Scale bars indicate 50 μm. F, Quantification of fibrotic area in infarcted and noninfarcted areas 28 days after MI in WT (n=10), IL-23-KO (n=9), and IL-17A-KO (n=11) mice. Statistical analysis was performed using 2-way ANOVA followed by Tukey's post hoc analysis (B) or Kruskall–Wallis tests with Dunn's multiple comparisons (D and F). LV indicates left ventricular; MI, myocardial infarction; WT, wild-type; and KO, knockout. *P<0.05, **P<0.01 vs WT heart.

Severe anterior MI induced by proximal left coronary artery ligation in mice leads to high mortality because of severe LV dysfunction. Some of mice die from cardiac rupture. The protective effect of IL-23 and IL-17A deficiency on survival became obvious after 7 days. The survival rate on day 28 post-MI was 34.7% (25/72) in WT mice, 65.5% (19/29) in IL-17A-KO mice, and 62.5% (15/24) in IL-23-KO mice (Figure 4A).

Survivors were evaluated for cardiac remodeling on day 28 post-MI. Echocardiographic examination revealed a markedly enlarged heart (LVEDD 6.47±0.25 mm, n=16) with reduced LV systolic function (FS 5.2±1.1%, n=16) in WT mice following MI (Figure 4B), whereas IL-17A-KO and IL-23-KO mice showed significantly less LV enlargement (LVEDD 5.64±0.21 mm, n=16, and 5.61±0.20 mm, n=9, respectively) and less severe LV dysfunction (FS 11.0±1.7%, n=16, and 10.5±1.7%, n=9, respectively). LVESP and maximum and minimum dP/dt, the index of contractility, were higher in IL-17A-KO and IL-23-KO mice compared with WT mice, whereas the ratio of heart to body weight was lower in both IL-17A-KO and IL-23-KO mice than in WT mice (Table 1). The infarct size (infarct circumference/LV circumference) was significantly smaller in IL-17A-KO (35.7±2.0%, n=16) and IL-23-KO (36.3±2.7%, n=9) mice compared with WT mice (46.3±2.3%, n=16) (Figure 4C and 4D). The area of myocardial fibrosis in noninfarcted heart was significantly smaller in IL-17A-KO (0.65±0.22%, n=11) and IL-23-KO (0.70±0.18%, n=9) mice than in WT mice (1.72±0.27%, n=10) (Figure 4E and 4F).

**Table 1.** Organ Weights and Hemodynamic Data 28 Days After MI

	Sham				MI			
		
	WT	IL-23 KO	IL-17A KO	TCRγδ KO	WT	IL-23 KO	IL-17A KO	TCRγδ KO
Organ weight								

n	5	6	6	6	16	10	16	10

BW, g	24.6±0.6	25.4±0.5	25.1±0.7	24.4±0.3	23.9±0.6	24.7±0.5	25.2±0.4	24.3±0.2

HW/BW, mg/g	4.52±0.23	4.47±0.31	4.55±0.16	4.61±0.33	6.56±0.28*	5.86±0.11*†	5.78±0.20*†	5.82±0.28*†

LW/BW, mg/g	4.79±0.22	4.82±0.18	4.69±0.24	4.87±0.28	7.12±0.43*	6.31±0.38*†	6.46±0.43*†	6.29±0.35*†

Hemodynamics								

n	5	6	6	6	8	9	11	7

HR, bpm	522.3±7.2	531.3±5.7	526.4±3.1	535.9±4.9	531.2±5.6	528.2±4.7	537±3.9	540.2±5.1

LVESP, mm Hg	107.6±1.8	105.6±2.1	103.4±1.6	102.8±1.2	75.2±3.7*	85.5±2.7*†	86.2±3.1*†	87.2±3.6*†

+dP/dt, mm Hg/s	11 090±1126	11 676±987	12 072±856	11 370±762	6352±642*	7687±425*†	7524±347*†	7852±431*†

−dP/dt, mm Hg/s	−8922±653	−9037±473	−8695±564	−8959±322	−4916±221*	−5501±194*†	−5323±305*	−5472±203*

Results are presented as mean±SEM. MI indicates myocardial infarction; WT, wild-type; KO, knockout; BW, body weight; HW, heart weight; LW, lung weight; HR, heart rate; bpm, beats per minute; LVESP, left ventricular end-systolic pressure.

* *P*<0.05 vs corresponding sham group.

† *P*<0.05 vs WT MI group (2-way ANOVA followed by Tukey's post hoc analysis).

### Macrophages and Neutrophils Were the Major Cellular Source of IL-23, Whereas γδT Cells Were the Major Cellular Source of IL-17A in Infarcted Heart

**Figure 5.**
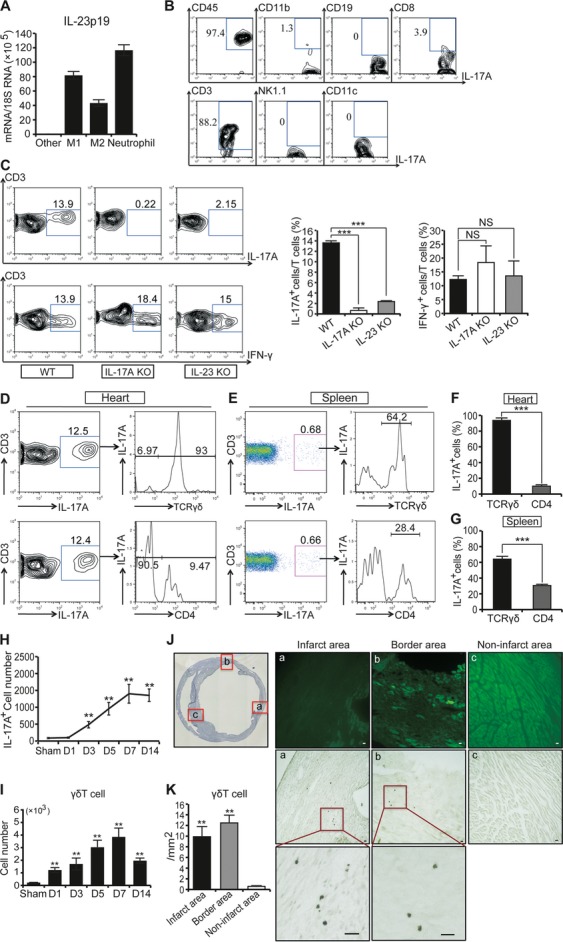
Major cellular sources of IL-23 and IL-17A. A, IL-23p19 mRNA expression in each cell population prepared from heart on day 1 post-MI (n=3). Other, CD11b^−^ cells; M_1_, M_1_ macrophage; M_2_, M_2_ macrophage. B, Intracellular cytokine and surface marker staining was performed on the enriched leukocytes prepared from heart on day 7 post-MI. Data are representative of 4 independent experiments. C, Comparison of IL-17A-producing and IFN-γ-producing cells in infiltrated T lymphocytes from infarcted heart on day 7 post-MI between WT and KO mice. Data are representative of 4 independent experiments. WT indicates wild-type; KO, knockout; MI, myocardial infarction; and NS, not significant; ***P<0.001 vs WT heart. D through G, IL-17A^+^ T-cell populations prepared from heart (D and F) and spleen (E and G) on day 7 post-MI were further analyzed for TCRγδ and CD4 expression by flow cytometry (n=4 each). ***P<0.001. H, Time course of change in numbers of infiltrating IL-17A^+^ cells in the infarcted heart (n=4 to 6 each). **P<0.01 vs sham heart. I, Quantities represent absolute number of γδT cells per heart (n=4 to 6 each). **P<0.01 vs sham. J, α-Actinin (green fluorescence, upper panel) and TCRγδ (middle and bottom panels) immunostaining of heart tissue on day 7 post-MI. K, Number of γδT cells in infarct, border, and noninfarct areas (n=5). **P<0.01 vs noninfarct area. Scale bar, 20 μm. Data in (F) and (G) were analyzed by Mann–Whitney *U* tests; data in (C), (H), (I), and (K) were analyzed by Kruskall–Wallis tests with Dunn's multiple comparisons.

We investigated the major cellular source of the respective cytokines in the infarcted hearts. The leukocyte-rich fraction collected from day 1 post-MI hearts was separated into CD45^+^CD11b^+^F4/80^+^ macrophages, CD45^+^CD11b^+^Ly-6G^+^ neutrophils, and other cells. The macrophages were further divided into 2 groups, CD206^low^ classically (M_1_) and CD206^high^ alternatively activated (M_2_). IL-23p19 mRNA expression was detected in the neutrophils and macrophages (M_1_>M_2_), but it was much lower in the other cell subsets (Figure 5A).

**Figure 6.**
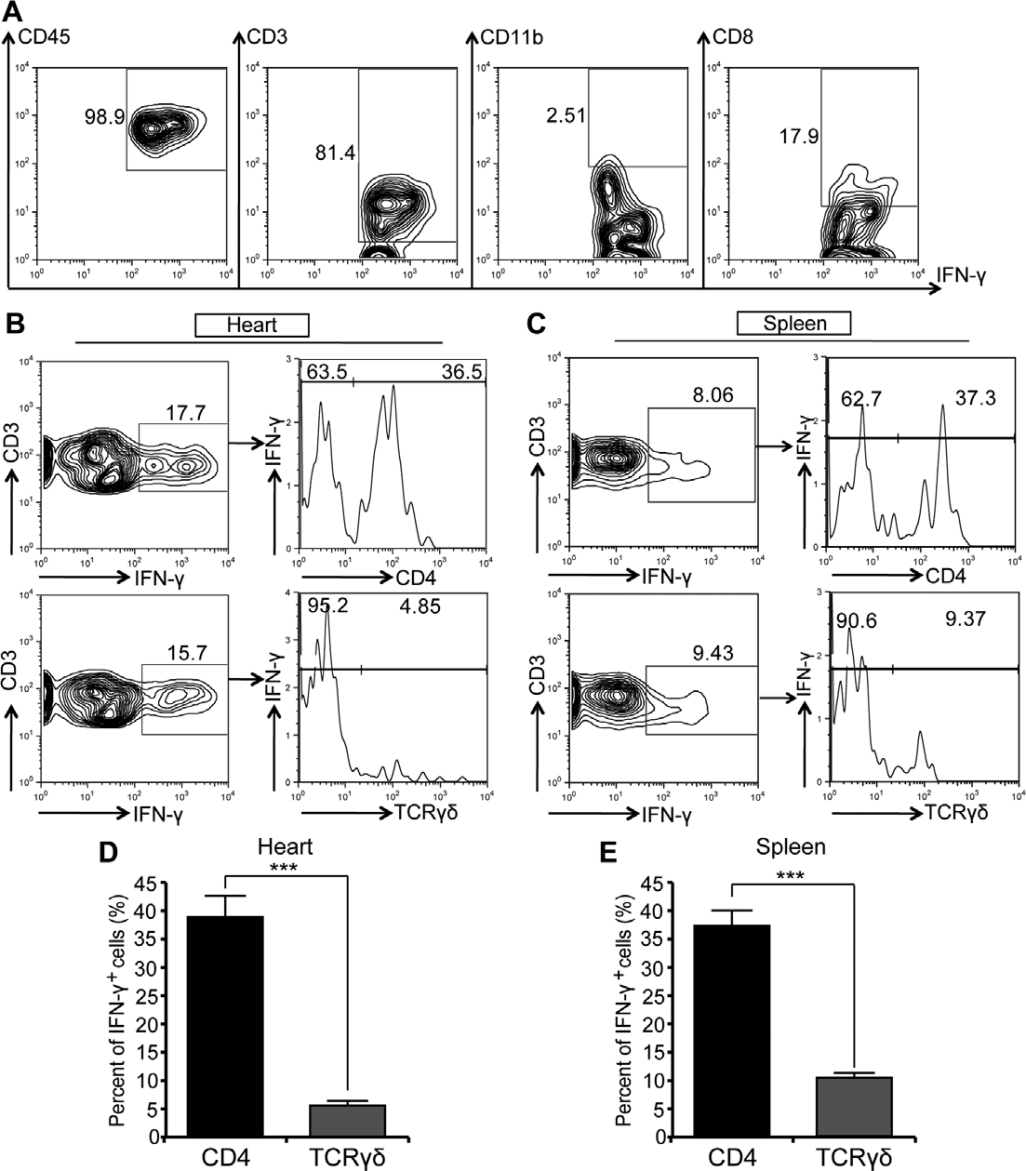
The cellular source of IFN-γ. A, Intracellular cytokine staining combined with surface markers was performed on the enriched leukocytes prepared from heart on day 7 post-MI. Data are representative of 4 independent experiments. IFN-γ^+^ T-cell populations prepared from heart (B and D) and spleen (C and E) on day 7 post-MI were further analyzed for TCRγδ and CD4 levels by flow cytometry (n=4). MI indicates myocardial infarction. ***P<0.001. Data in (D) and (E) were analyzed by Mann–Whitney *U* tests.

Intracellular cytokine staining revealed that 90.1±1.2% of IL-17A-expressing cells in the infarcted hearts on day 7 post-MI were CD3^+^ T lymphocytes (Figure 5B). IL-23-KO mice had a significantly smaller proportion of IL-17A-producing cells among CD3^+^ T lymphocytes compared with WT mice (13.6±0.4% versus 2.2±0.2%, n=4, *P*<0.001), whereas the proportion of IFN-γ-producing cells among CD3^+^ T lymphocytes was not affected in IL-17A-KO or IL-23-KO mice (Figure 5C). More than 90% of IL-17A-producing T cells in this study were CD4^−^ TCRγδ^+^ (γδT) cells, but not CD4^+^ T cells (Th17) (Figure 5D and 5F). By contrast, both CD4^−^ TCRγδ^+^ cells (65.2±2.6%) and CD4^+^ T cells (29.6±1.7%) were predominant cellular sources of IL-17A in the spleen (Figure 5E and 5G). After MI, the number of IL-17A-expressing cells gradually increased to a peak 7 days post-MI and then remained high up to 14 days post-MI (Figure 5H). The γδT cells started to increase on day 1 and peaked on day 7 after MI (Figure 5I). Immunohistochemical examination revealed that γδT cells were mainly in the infarct and border areas, but not in the noninfarct area (Figure 5J and 5K). In addition, TCRγδ^+^ cells were not major sources of IFN-γ in the infarcted heart, but showed a relatively higher contribution of IFN-γ production in spleen (5% in heart versus 10% in spleen), thus splenic γδT cells produced both IL-17A and IFN-γ (Figure 6A through 6E).

### IL-23 Enhanced γδT Cell Recruitment, Whereas IL-17A Promoted Neutrophil Infiltration Into Post-MI Heart

**Figure 7.**
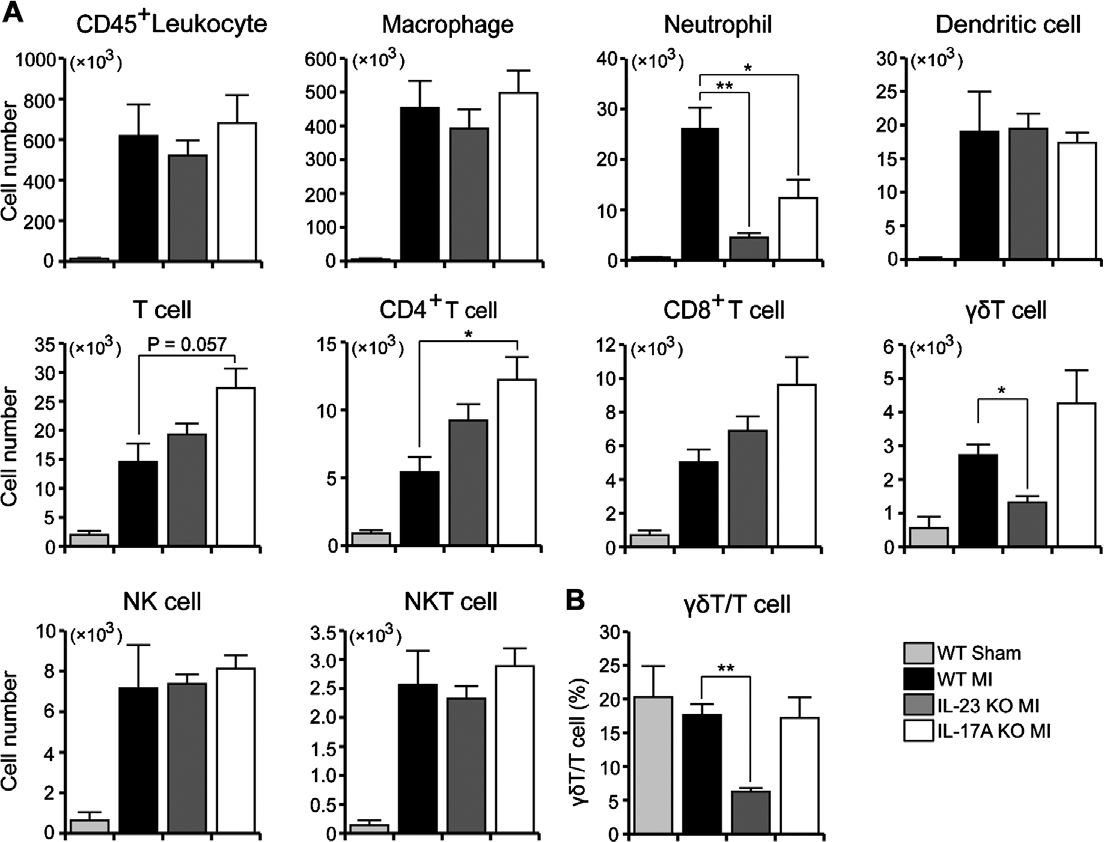
Comparison of immune cells infiltrated into infarcted heart among WT, IL-23-KO, and IL-17A-KO mice. A, Flow-cytometric analysis of infiltrating immune cell numbers in infarcted heart on day 7 post-MI between WT and KO mice (n=4 to 6 each). WT indicates wild-type; KO, knockout; and MI, myocardial infarction. *P<0.05, **P<0.01 vs WT MI. B, Percentage of γδT cells among T cells in infarcted heart on day 7 post-MI in WT and KO mice (n=4 to 6 each). **P<0.01 vs WT MI. Data in (A) and (B) were analyzed by Kruskall–Wallis tests with Dunn's multiple comparisons.

We analyzed the impact of deficiency in IL-23 or IL-17A on the cellular infiltrate in the infarcted hearts on day 7 post-MI. The total number of infiltrating CD45^+^ immune cells was not altered in IL-23-KO and IL-17A-KO mice compared with WT mice (Figure 7A). However, when we looked at leukocyte subsets, the number of infiltrating neutrophils was significantly reduced in both IL-23-KO and IL-17A-KO mice compared with WT mice. The number of infiltrating macrophages, DCs, NK cells, and NKT cells was not altered in IL-23-KO and IL-17A-KO mice compared with those in WT mice. Unexpectedly, the number of infiltrating CD4^+^ and CD8^+^ cells in the infarcted heart was higher in IL-23-KO and IL-17A-KO than in WT mice. Notably, the number of infiltrating γδT cells was markedly reduced in IL-23-KO mice but was not altered in IL-17A-KO mice compared with wild types (Figure 7A and 7B).

### IL-23 and IL-17A Affected Matrix Metalloproteinase and Fibrosis-Related Gene Expression in Infarcted Heart on Day 7 Post-MI, But Not on Day 2 Post-MI

**Figure 8.**
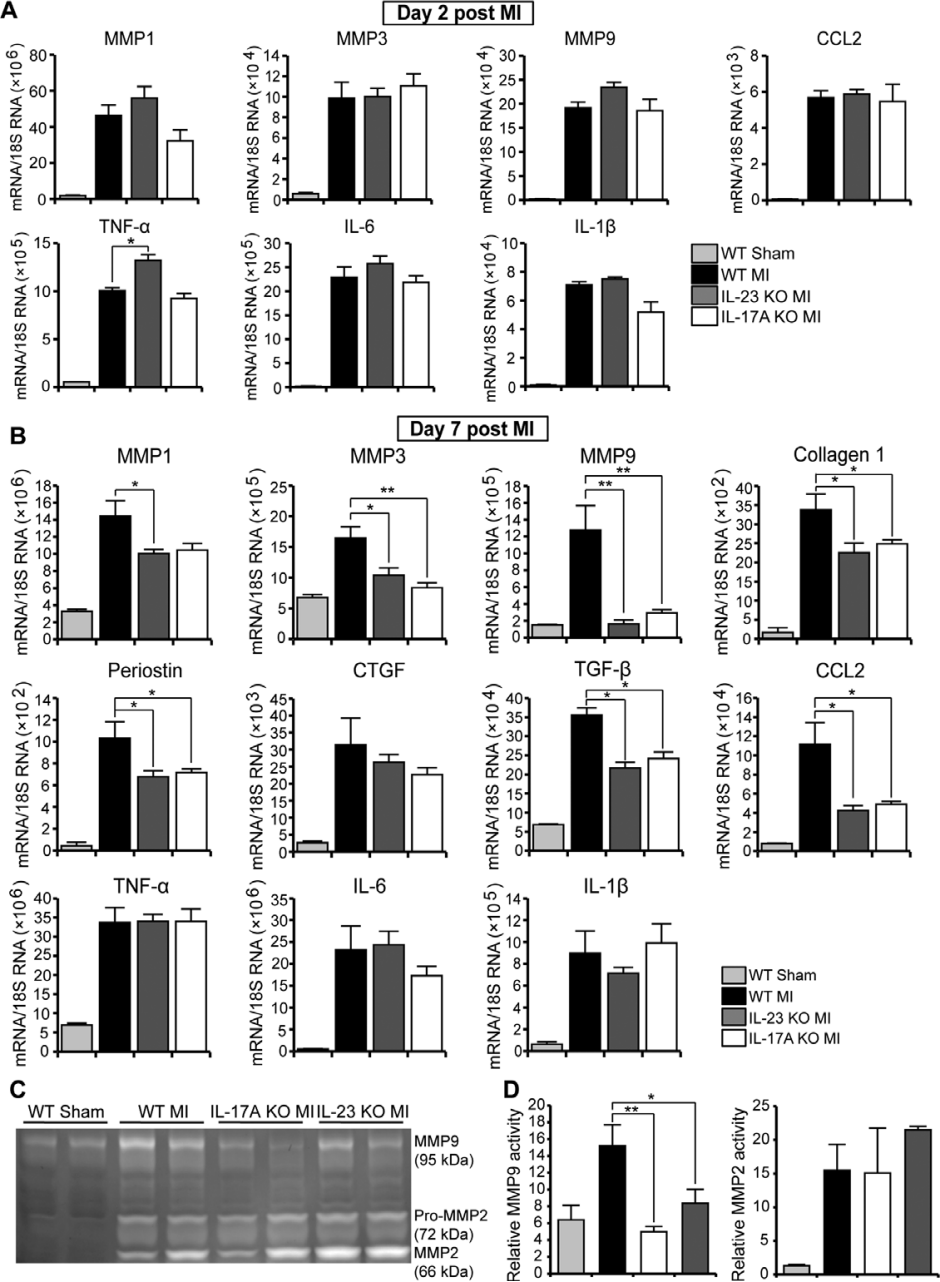
Comparison of fibrosis-related genes and inflammatory mediator expression in infarcted hearts of WT, IL-23-KO, and IL-17A-KO mice. A, Relative changes in levels of mRNA encoding MMPs, fibrosis-related genes, and proinflammatory cytokines measured by quantitative RT-PCR in heart tissue on day 2 post-MI (n=4 each). *P<0.05 vs WT MI. B, Relative changes in levels of mRNA encoding MMPs, fibrosis-related genes, and proinflammatory cytokines in heart tissue on day 7 post-MI (n=4 each). *P<0.05, **P<0.01 vs WT MI. C, Representative photograph of zymographic gel demonstrating MMP9 and MMP2 activities in heart tissues of WT and KO mice on day 7 after MI. D, Quantitative analysis of MMP9 and MMP2 activities after MI based on gelatin zymography. Data were obtained from 3 independent experiments. *P<0.05, **P<0.01 vs WT MI. MMP indicates matrix metalloproteinases; WT, wild-type; KO, knockout; and MI, myocardial infarction. Data in (A), (B), and (D) were analyzed by Kruskall–Wallis tests with Dunn's multiple comparisons.

On day 2 post MI, expression of matrix metalloproteinases (MMP) 1, *MMP3, MMP9, CCL2, IL-6,* and *IL-1β* mRNA was not altered in IL-23-KO and IL-17A-KO mice compared with WT mice, whereas TNF-α expression was slightly higher in IL-23-KO mice (Figure 8A). In contrast, on day 7 post MI, expression of *MMP1, MMP3,* and *MMP9* mRNA was significantly lower in both IL-23-KO and IL-17A-KO mice compared with that in WT mice, as was the mRNA expression of the fibrosis-related genes collagen 1, periostin, and TGF-β (Figure 8B). Consistent with this, MMP9 activity as assessed by gelatin zymography was significantly suppressed in both IL-23-KO and IL-17A-KO mice compared with WT mice (Figure 8C and 8D). mRNA expression of CCL2, a chemokine that mediates monocyte/macrophage recruitment, was significantly lower in the IL-23-KO and IL-17A-KO than in the WT mice. Expression of TNF-α, IL-6, and IL-1β mRNA was not altered in IL-23-KO and IL-17A-KO mice compared with that in WT mice (Figure 8B).

### γδT Cells Contributed to Cardiac Remodeling After Myocardial Infarction

**Figure 9.**
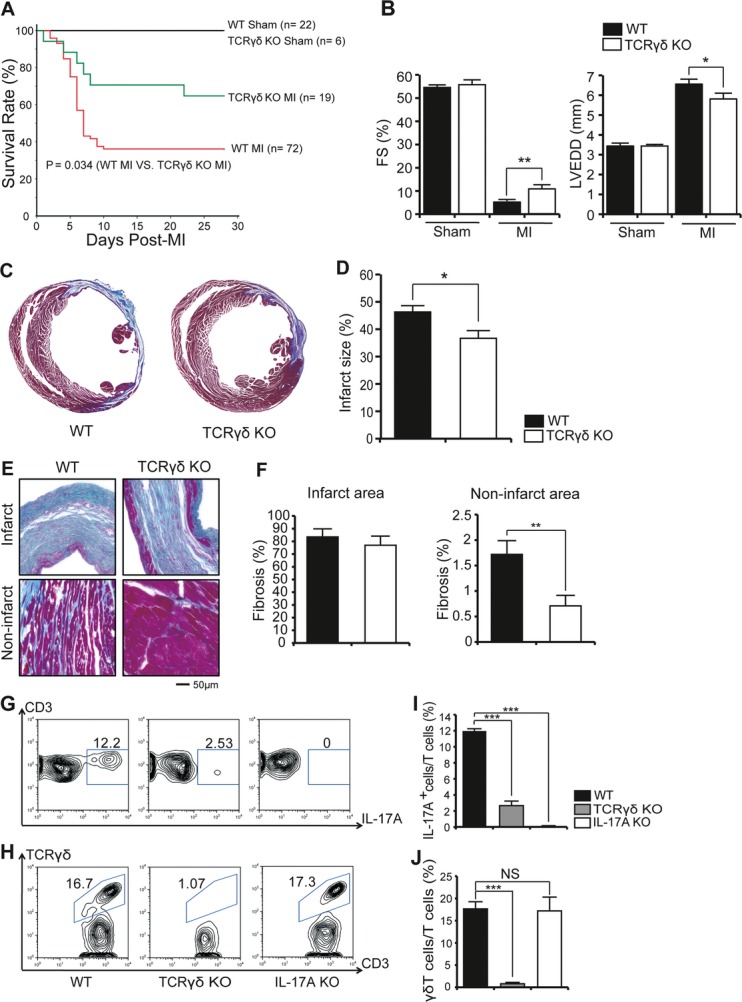
Deficiency in TCRγδ conferred resistance to LV remodeling on day 28 post-MI. Kaplan–Meier survival analysis (A) and echocardiographic analysis (B) in WT and TCRγδ-KO mice (n=10 to 16 each). *P<0.05, **P<0.01 vs WT MI (2-way ANOVA followed by Tukey's post hoc analysis). C, Azan staining of cardiac sections in WT and TCRγδ-KO mice after MI. D, Infarct size determined by Azan staining of sections (n=10 to 16). *P<0.05 vs WT MI. E, Representative Azan-stained images of infarcted and noninfarcted areas 28 days after MI; blue staining indicates fibrosis. Scale bars indicate 50 μm. F, Quantification of fibrotic area in infarcted and noninfarcted areas 28 days after MI in WT (n=10) and TCRγδ-KO (n=9) mice. **P<0.01 vs WT heart. Data in (D) and (F) were analyzed by Mann–Whitney *U* tests. G through J, Comparison of IL-17A-producing T cells (G and I) and γδT cells (H and J) in infiltrating T lymphocytes in infarcted heart on day 7 post-MI between WT and KO mice (n=4 each). WT indicates wild-type; KO, knockout; MI, myocardial infarction; and LV, left ventricular. ***P<0.001 vs WT MI (Kruskall–Wallis tests with Dunn's multiple comparisons).

We investigated the pathogenic importance of the demonstrated γδT cell response in post-MI remodeling. When we subjected TCRγδ-deficient (TCRγδ-KO) mice to MI, infarct size and LV dysfunction on day 1 post MI were similar to those of WT mice (Figure 3A through 3C). However, the survival rate on day 28 post-MI was significantly improved in TCRγδ-KO mice (63.2% [12/19]) compared with that in WT mice (34.7% [25/72]) (Figure 9A), and this protective effect of γδT cell deficiency on survival became obvious after 7 days.

The post-MI LV remodeling in TCRγδ-KO mice on day 28 post-MI was significantly attenuated compared with that in WT mice, as was LV enlargement and the severity of LV dysfunction (LVEDD 6.56±0.24 versus 5.71±0.21 mm, FS 5.2±1.1% versus 10.7±1.8%, n=10 to 16) (Figure 9B). LVESP and maximum and minimum dP/dt were higher, whereas the heart weight/body weight ratio was lower in TCRγδ-KO mice compared with WT mice (Table 1). Azan staining revealed a reduced infarct size (infarct circumference/LV circumference) in TCRγδ-KO hearts compared with WT mice (46.3±2.3% versus 36.7±2.8%, n=10 to 16) (Figure 9C and 9D), and the area of myocardial fibrosis in noninfarcted heart was significantly smaller in TCRγδ-KO compared with WT mice (0.71±0.21%, n=9, versus 1.72±0.27%, n=10) (Figure 9E and 9F).

**Figure 10.**
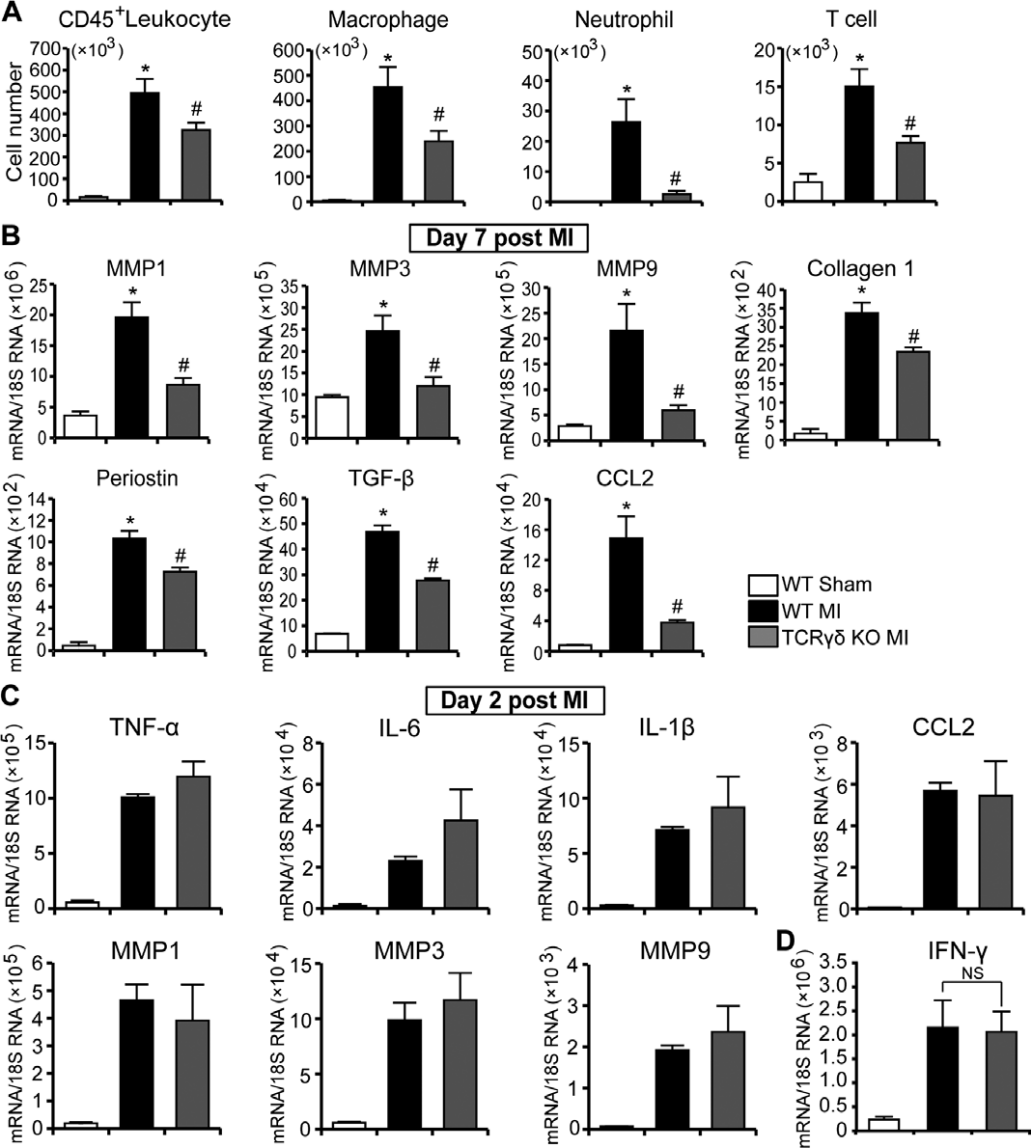
Effect of γδT cell deficiency on immune cell infiltration and inflammatory mediator expression. A, Flow-cytometric analysis of infiltrating immune cells in the heart on day 7 post-MI between WT and KO mice (n=4 each). *P<0.05 vs WT sham; #*P*<0.05 vs WT MI. B, Relative changes in levels of mRNA encoding MMPs, fibrosis-related genes, and chemokines in heart tissue on day 7 post-MI (n=4 each). *P<0.05 vs WT sham; #P<0.05 vs WT MI. C, mRNA levels of indicated genes were measured by quantitative RT-PCR in heart tissue on day 2 post-MI (n=4 each). D, IFN-γ mRNA levels were measured by quantitative RT-PCR in heart tissue on day 7 post-MI (n=4 each). MMP indicates matrix metalloproteinases; WT, wild-type; KO, knockout; and MI, myocardial infarction. Data in (A), (B), (C), and (D) were analyzed by Kruskall–Wallis tests with Dunn's multiple comparisons.

The TCRγδ-KO mice showed markedly lower numbers of infiltrating IL-17A-expressing T cells in infarcted hearts on day 7 post-MI compared with WT mice (12.1±0.3% versus 2.5±0.6%, n=4) (Figure 9G and 9I). In contrast, the number of infiltrating γδT cells in IL-17A-KO mice was comparable to that in WT mice in day 7 post-MI heart (Figure 9H and 9J), but there were no IL-17A-expressing cells. Furthermore, TCRγδ-KO mice had a markedly decreased number of CD45^+^-leukocytes including macrophages, T cells, and neutrophils than WT mice (Figure 10A). MMP1, MMP3, MMP9, collagen 1, periostin, TGF-β, and CCL2 were markedly attenuated on day 7 in TCRγδ-KO mice compared with WT mice (Figure 10B), whereas MMP1, MMP3, MMP9, TNF-α, IL-1β, IL-6, and CCL2 expression was comparable between the groups on day 2 post-MI (Figure 10C). Expression of IFN-γ mRNA on day 7 post-MI did not differ between TCRγδ-KO mice and WT mice (Figure 10D).

### Sphingosine-1-Phosphate Receptor and CCL20/CCR6 Signaling Pathways Mediated γδT Cell Recruitment to Infarcted Heart

**Figure 11.**
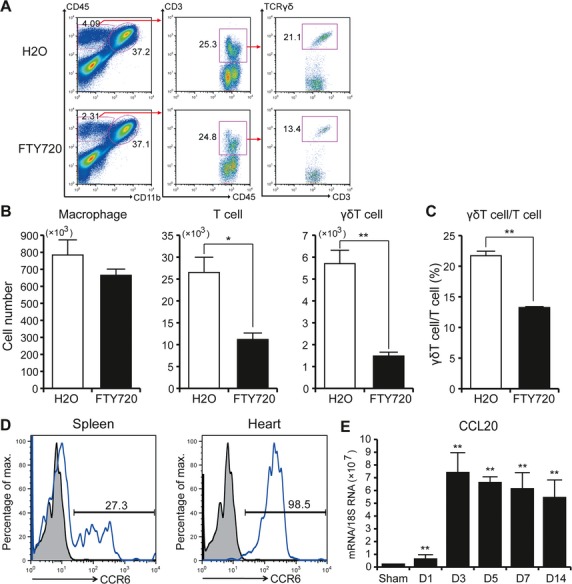
Sphingosine 1-phosphate (S1P) signaling and the CCL20/CCR6 axis mediate γδT cell recruitment into the heart. A through C, FTY720 or H_2_O was administered before MI and every 24 hours thereafter. A, Representative figures revealed that proportions of the lymphocyte (CD45^+^CD11b^−^) and γδT-cell (CD45^+^CD11b^−^TCRγδ^+^) infiltration into the heart were suppressed by FTY720 on day 6 after MI. B and C, Absolute number of heart-infiltrating inflammatory cells (B) and percentage of γδT cells among total T cells (C) were quantified on day 6 after MI (n=6 for H_2_O group and n=4 for FTY720 group). *P<0.05, **P<0.01 vs H_2_O group. Data in (B) and (C) were analyzed by Mann–Whitney *U* tests. D, CCR6 expression in splenic and cardiac γδT cells on day 7 after MI. Gray histogram indicates isoform controls. Data are representative of 3 experiments. E, Time course of changes in mRNA expression of CCL20 in post-MI heart. Values were normalized to 18S (n=4 to 6 each). MI indicates myocardial infarction. **P<0.01 vs sham (Kruskall–Wallis tests with Dunn's multiple comparisons).

The immunomodulatory drug FTY720 interferes with sphingosine-1-phosphate (S1P) receptor signaling, leading to sequestration of lymphocytes in lymph nodes but not spleen.^30^ Infusion of FTY720 significantly suppressed the number of infiltrating T cells, and particularly γδT cells, in the postinfarct heart (Figure 11A through 11C). These findings indicated that cardiac γδT cells migrated from lymph nodes, at least in part via S1P receptor signaling.

Chemokine receptor 6 (CCR6) contributes to the migration of Th17 cells to a particular site of inflammation.^31^ We found that almost all cardiac γδT cells constitutively expressed CCR6, whereas only 27% of spleen γδT cells expressed CCR6 (Figure 11D). Expression of the CCR6 ligand CCL20 was significantly increased on day 1 post-MI, before peaking on day 3 post-MI, and then remaining high to day 14 post-MI (Figure 11E). These findings suggested an important role for the CCL20–CCR6 signaling axis in recruiting γδT cells into infarcted heart.

### Toll-Like Receptor (TLR) Signaling and IL-1β Worked in Concert With IL-23 to Drive IL-17A Production by Cardiac γδT Cells

**Figure 12.**
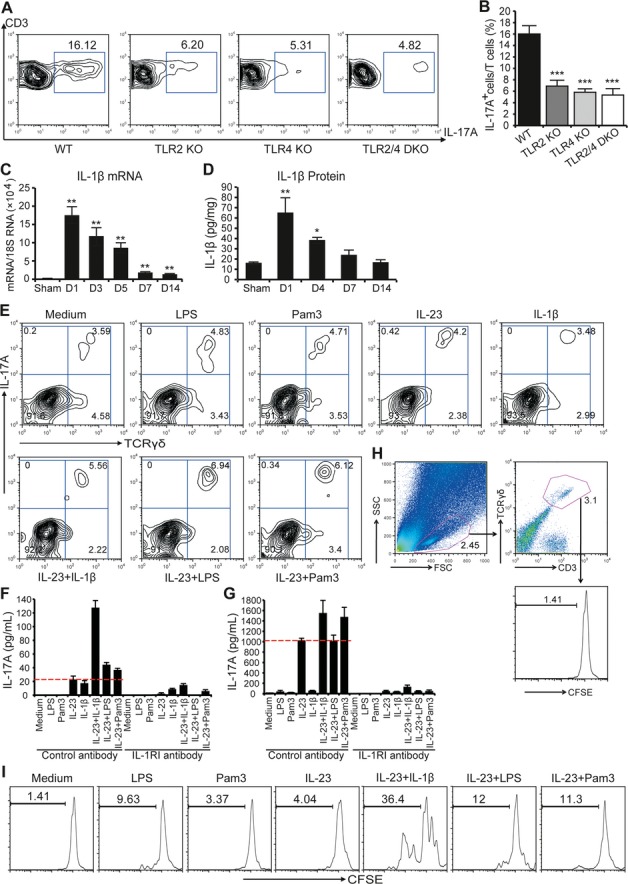
TLR signaling and IL-1β are involved in IL-23-induced IL-17A production from γδT cells. A, Comparison of IL-17A-producing infiltrating T lymphocytes isolated from infarcted hearts on day 7 post-MI among WT, TLR2-KO, TLR4-KO, and TLR2/4-DKO mice. Data are representative of 4 independent experiments. B, Percentage of IL-17A^+^ cells among total T cells in the heart was quantified on day 7 after MI (n=4 to 7). **P<0.001 vs WT heart. C, Time course of changes in mRNA expression of IL-1β in heart tissue after MI. Values were normalized to 18S (n=4 to 6 each). **P<0.01 vs sham. D, IL-1β protein levels were measured by ELISA in left ventricular tissue after MI. Values were normalized to total protein concentration in left ventricular tissue (n=4 each). *P<0.05, **P<0.01 vs sham. E, Cardiac cells prepared from day 7 post-MI hearts were stimulated with IL-23, IL-1β, LPS, Pam3CSK4 (Pam3) alone, or IL-23 plus IL-1β and different pathogenic products for 3 days. Intracellular IL-17A production was determined by flow cytometry, gated on CD3^+^ T cells. Data are representative of 3 independent experiments. F and G, Cardiac total cells (F) and CD45^+^ cells (G) sorted from day 7 post-MI hearts were stimulated with the indicated cytokines and/or pathogenic products for 3 days either in the presence or the absence of IL-1RI antibody. Supernatants were then harvested and measured for IL-17A by ELISA (n=4 for each group). H and I, Cardiac cell suspensions prepared from day 7 post-MI heart were labeled with CFSE and then stimulated with the indicated cytokines and/or pathogenic products for 3 days. Cells were harvested and stained with anti-CD3 and anti-TCRγδ antibodies. Cells were gated on γδT cells for flow cytometry. Representative gating strategy for γδT cell proliferation assay (H) and CFSE dilution assay (I) is shown. WT indicates wild-type; Data are representative of 3 independent experiments. Data in (B), (C), and (D) were analyzed by Kruskall–Wallis tests with Dunn's multiple comparisons.

Engagement of these receptors with their respective ligands could stimulate NF-κB and IL-23 production. TLR1 and TLR2 were also expressed in γδT cells.^32^ To examine whether TLR2 and TLR4 operate upstream of IL-17A production in the infarcted heart, we analyzed the number of IL-17A-expressing T cells in day 7 post-MI heart from WT, TLR2-KO, TLR4-KO, or TLR2/4-DKO mice using flow cytometry. TLR2-KO, TLR4-KO, and TLR2/4-DKO mice had a significantly smaller proportion of IL-17A-producing cells among CD3^+^ T lymphocytes than did WT mice (15.9±1.4% versus 6.2±0.9%, 5.2±0.5%, and 4.8±1.0%, respectively, n=4 to 7; *P*<0.001) (Figure 12A and 12B). IL-1β mRNA and protein levels in the heart were also dramatically increased as early as 24 hours after MI and remained significantly elevated above baseline levels thereafter (Figure 2C and 2D). Therefore, we examined the synergistic effect of TLRs ligands and IL-1β acting with IL-23 on the proliferation of and IL-17A production from γδT cells. Cardiac cells prepared from day 7 post-MI hearts were stimulated using lipopolysaccharide (LPS; TLR4 ligand), Pam3CSK4 (TLR1/2 ligand), and IL-1β, each alone and in combination with IL-23. A synergistic effect on IL-17A production was observed when cells were exposed to the combination of IL-23 and LPS or of Pam3CSK4 and IL-1β (Figure 12E and 12F). The synergistic effect of TLR ligands and IL-1β on IL-17A production was further examined using CD45^+^ cells sorted from day 7 post-MI hearts. LPS, Pam3CSK4, or IL-1β alone could not stimulate IL-17A production; however, IL-23 alone was sufficient to drive IL-17A production. A synergistic effect on IL-17A production was observed when CD45^+^ cells were exposed to the combination of IL-23 and IL-1β or of IL-23 and Pam3CSK4, but not to the combination of IL-23 and LPS (Figure 12G). Similarly, IL-17A production from cardiac cells or CD45^+^ cells was significantly suppressed when cells were pretreated with IL-1 receptor I antibody (IL-1RI) (Figure 12F and 12G), suggesting that the IL-1R signaling pathway is essential for IL-17A production in heart.

Examination of the proliferation-promoting effect of LPS, Pam3CSK4, and IL-1β on γδT cells by the CFSE dilution assay showed that the combination of IL-23 and IL-1β had the most pronounced effect on cardiac γδT cell proliferation (Figure 12H and 12I).

### IL-17A Had Proapoptotic, Profibrotic, or Proinflammatory Properties

**Figure 13.**
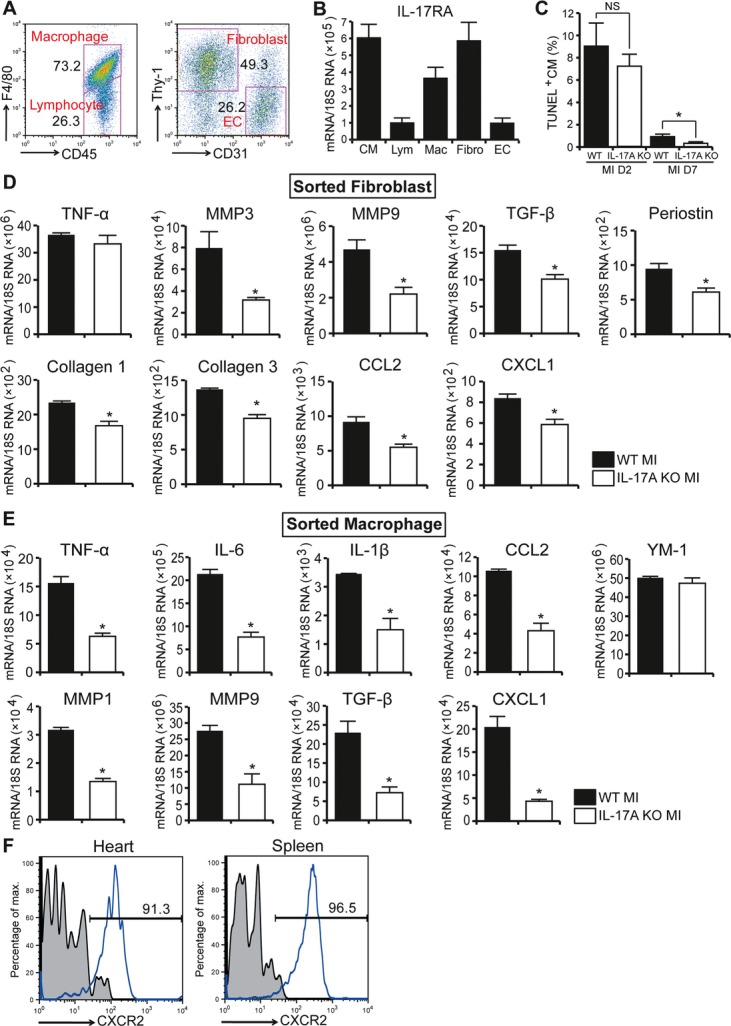
In vivo cell-specific function of IL-17RA in the infarcted heart. A, Single-cell suspensions isolated from heart on day 7 post-MI were sorted by flow cytometry. Macrophages (Mac): CD45^+^F4/80^+^; lymphocytes (Lym): CD45^+^F4/80^−^; fibroblasts (Fibro): CD45^−^Thy-1^+^CD31^−^; endothelial cells (EC): CD45^−^CD31^+^Thy-1^−^. B, IL-17RA expression in each fraction (n=3). C, Apoptotic cardiomyocytes were detected by TUNEL staining combined with α-actinin staining in the border zone on day 2 and day 7 after MI in WT and IL-17A-KO mice (n=6 each). NS indicates not significant; WT, wild-type; MI, myocardial infarction; MMP, matrix metalloproteinases; and KO, knockout. *P<0.05 (2-way ANOVA followed by Tukey's post hoc analysis). D, mRNA levels of indicated genes in sorted fibroblasts (n=5). *P<0.05 vs WT. E, mRNA levels of indicated genes in sorted macrophages (n=5 each). *P<0.05 vs WT. F, CXCR2 expression in splenic and cardiac neutrophils on day 7 after MI. Gray histogram indicates isoform controls. Data are representative of 3 experiments. Data in (D) and (E) were analyzed by Mann–Whitney *U* tests.

Finally, we investigated the mechanisms behind the pathogenic role of IL-17A in post-MI cardiac remodeling. To identify the important cellular targets for IL-17A in vivo, we separated macrophages, lymphocytes, fibroblasts, endothelial cells, and cardiomyocytes from the infarcted hearts on day 7 post-MI. IL-17RA was highly expressed in cardiomyocytes, fibroblasts, and macrophages (Figure 13A and 13B).

**Figure 14.**
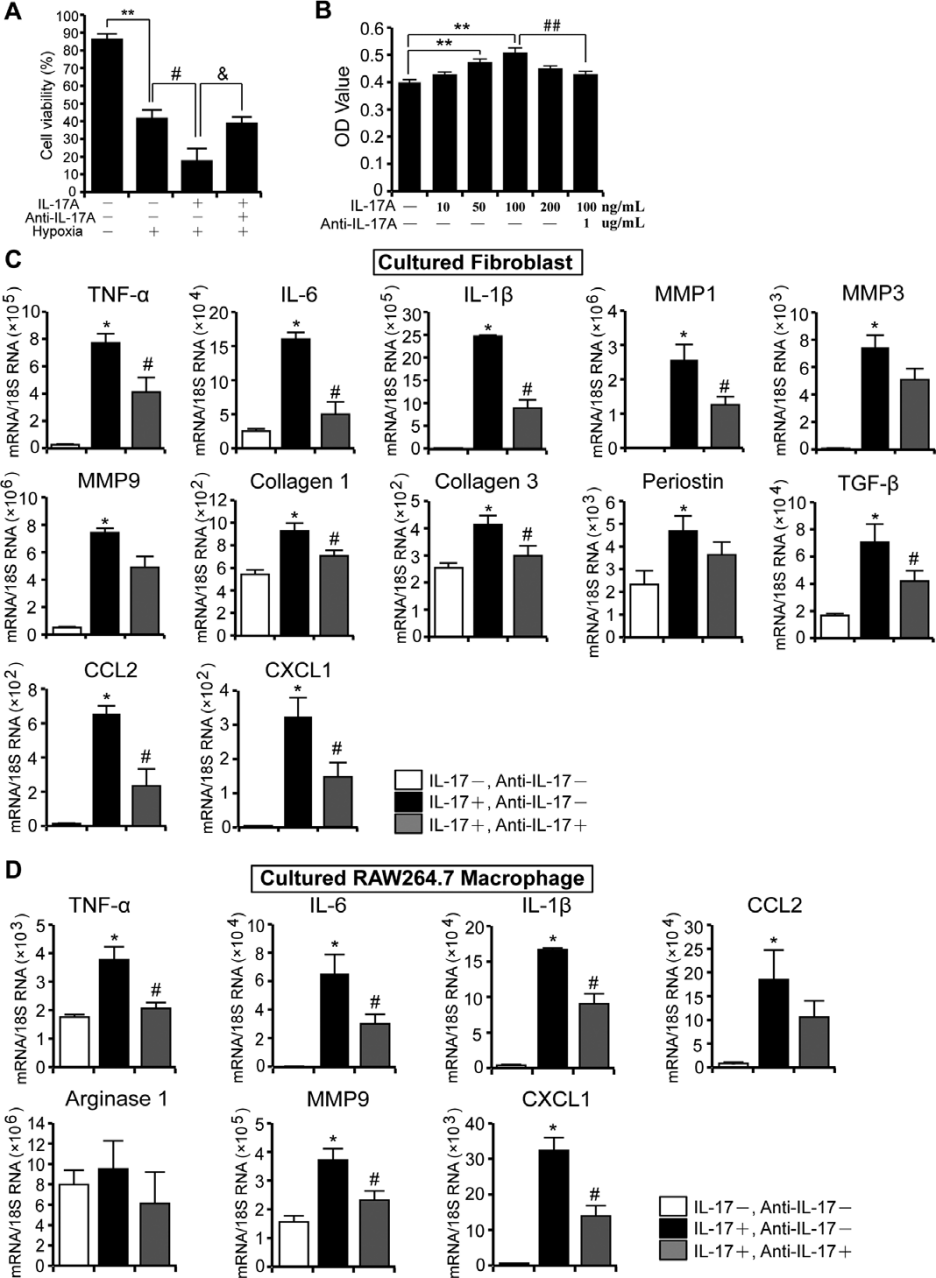
Effect of IL-17A on cultured cardiomyocytes, fibroblasts and RAW264.7 macrophages. A, Neonatal mouse cardiomyocytes were pretreated with neutralizing anti-IL-17A antibody (1 μg/mL) followed by hypoxia and then IL-17A (100 ng/mL) stimulation. Dead cells and viable cells were determined as described in Methods. Viable cells were quantified by counting 100 cells in 5 independent experiments (n=5). **P<0.01, #*P*<0.05, and &*P*<0.05. B, Neonatal mouse fibroblasts were stimulated with IL-17A at the indicated concentrations for 72 hours in the presence or absence of neutralizing anti-IL-17A antibody, and cell proliferation was measured with a cell counting kit-8 (n=6 each). **P<0.01 vs vehicle-treated cells, ##*P*<0.01 vs anti-IL-17A antibody–untreated cells. C, Neonatal mouse fibroblasts were stimulated with IL-17A (100 ng/mL) for 24 hours in the presence or absence of neutralizing anti-IL-17A antibody (1 μg/mL), and mRNA levels of indicated genes were measured by real-time PCR (n=5 each). *P<0.05 vs vehicle-treated cells, #*P*<0.05 vs anti-IL-17A antibody–untreated cells. D, RAW264.7 macrophages were stimulated with IL-17A (100 ng/mL) for 24 hours in the presence or absence of neutralizing anti-IL-17A antibody (1 μg/mL), and mRNA levels of indicated genes were measured by real-time PCR (n=5 each). *P<0.05 vs vehicle-treated cells, #*P*<0.05 vs anti-IL-17A antibody–untreated cells. MMP indicates matrix metalloproteinases. Data in (A), (B), (C), and (D) were analyzed by Kruskall–Wallis tests with Dunn's multiple comparisons.

To examine the impact of IL-17A on cardiomyocyte survival, we performed TUNEL staining combined with α-actinin staining on the infarcted hearts of WT and IL-17A-KO mice on both day 2 and day 7 post-MI. Ablation of IL-17A had no effect on the number of TUNEL-positive cardiomyocytes in the border regions on day 2 (9.0±2.1% and 6.8±1.2%, respectively, n=6). Although there were markedly fewer TUNEL-positive cardiomyocytes on day 7 post-MI than on day 2 in WT mice, ablation of IL-17A further decreased the number of survivors (WT, 1.2±0.2%, versus IL-17A-KO, 0.5±0.1%, n=6) (Figure 13C). Consistent with previous reports,^22,23^ IL-17A significantly augmented low serum/hypoxia-induced cell death in cultured murine cardiomyocytes, and this cytotoxic effect of IL-17A could be completely rescued by neutralizing anti-IL-17A antibody (Figure 14A).

Next we investigated the effect of IL-17A on cardiac fibroblasts. Real-time PCR analysis revealed that sorted fibroblasts from the infarcted hearts on day 7 post-MI had significantly lower mRNA levels of *MMP3, MMP9,* profibrotic genes (*TGF-β*, collagen 1, collagen 3, and periostin), and chemokines (CCL2, CXCL1) in IL-17A-KO mice compared with WT mice (Figure 13D). Expression of TNF-α was not altered. In culture, IL-17A increased murine cardiac fibroblast proliferation in a dose-dependent manner, and this proliferative effect of IL-17A was completely abolished by neutralizing anti-IL-17A antibody (Figure 14B). Furthermore, IL-17A stimulated the expression of proinflammatory cytokines (TNF-α, IL-6, and IL-β), MMPs (MMP1, -3, -9), profibrotic genes (*TGF-β*, collagen 1, collagen 3, and periostin), and chemokines (CCL2, CXCL1) in cultured cardiac fibroblasts, whereas almost all this IL-17A-stimulated gene induction was significantly abrogated by neutralizing anti-IL-17A antibody (Figure 14C).

We also examined the gene expression profiles in macrophages isolated from the infarcted hearts on day 7 post-MI (Figure 13E). Expression of M_1_ macrophage signature genes such as *TNF-α, IL-6, IL-1β, CCL2*, and *CXCL1* in the sorted macrophages were dramatically suppressed in the IL-17A-KO mice compared with WT mice, while those of M_2_ signature gene YM-1 was not altered. The mRNA levels of *MMP1, MMP9,* and *TGF-β* in the sorted macrophages were also lower in the IL-17A-KO mice than in WT cells. In the cultured monocyte/macrophage cell line RAW264.7, IL-17A significantly increased mRNA expression of M_1_ macrophage signature genes, such as *TNF-α, IL-6, IL-1β, CCL2, MMP9,* and *CXCL1*, but did not alter those of the M_2_ signature gene arginase-1 (Figure 14D), and these gene inductions in RAW264.7 cells were blocked by neutralizing anti-IL-17A antibody. The above results strongly suggested that IL-17A has proapoptotic, profibrotic, and proinflammatory properties that combine to promote LV remodeling.

## Discussion

Two recent articles demonstrated that IL-17A is involved in the early cardiomyocyte death evident in ischemia–reperfusion injury, with functional involvement demonstrated 2 to 3 hours after reperfusion.^22,23^ Here, we created a substantially sized MI by permanent ligation of the coronary artery, and identified a functional link between the IL-23/IL-17A signaling axis and γδT cells in late-stage LV remodeling after MI. Despite the finding that infarct size 24 hours after surgery was comparable among all mice including WT, ablation of IL-23, IL-17A, or γδT cells improved survival after 7 days, limited infarct expansion, and reduced fibrosis in the noninfarcted myocardium, alleviating LV dilatation and systolic dysfunction on day 28 post-MI. IL-17A was not involved in the acute inflammatory response on day 2 post-MI.

The number of IL-17A-expressing cells gradually increased after MI to a peak 7 days post-MI and then remained high to 14 days post-MI. This trend was consistent with the time course change in the numbers of infiltrating γδT cells in infarcted heart. The γδT cells constituted approximately 90% of IL-17A-producing cells in the heart on day 7 post-MI. Furthermore, the infiltrating IL-17A-expressing T cells on day 7 post-MI were almost completely ablated in TCRγδ-KO mice. These results implicate the functional significance of cardiac γδT cells as a source of IL-17A after MI. Defining γδT cells as the major producer of IL-17A in infarcted heart coupled with the decreased number of neutrophils, macrophages, and T cells in the infarcted hearts of TCRγδ-KO mice, but not IL-17A-KO mice, on day 7 post-MI suggested that factors other than IL-17A also mediated the γδT cell-stimulated adverse cardiac remodeling.

**Figure 15.**
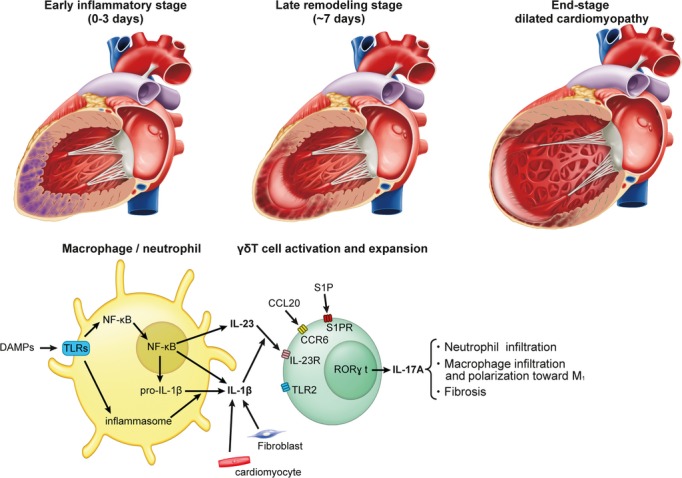
Mechanism of signal convergence on cardiac γδT cells for recruitment into infarcted heart and production of IL-17A. Damaged cells release DAMPs, which are recognized by TLRs. The subsequent TLR signaling promotes nuclear translocation of NF-κB, leading to expression of cytokines including pro-IL-1β and IL-23, which are indispensable for IL-17A production in γδT cells. Inflammasome activation leads to processing of caspase-1 into its enzymatically active form. Caspase-1 in turn cleaves pro-IL-1β, releasing active IL-1β, which can work in concert with IL-23 to drive IL-17A production in γδT cells, which also directly respond to TLR stimulation in synergy with IL-23. FTY720-sensitive S1P receptor signaling helps to mediate γδT-cell egress from lymph nodes, and CCR6-expressing γδT cells are recruited to the sites of injury that express CCL20. DAMPs indicates Danger associated molecular patterns; TLR, toll-like receptor.

We further provided mechanistic insight about how signals converge on the cardiac γδT cells to produce IL-17A. Danger-associated molecular patterns (DAMPs) such as mitochondrial DNA, adenosine triphosphate (ATP), high-mobility group box-1 (HMGB1), heat-shock protein (HSP), peroxiredoxins, and F-actin are detected via cell-surface TLRs or DNGR-1 and promote nuclear factor (NF)-κB nuclear translocation.^25,33–37^ NF-κB mediates expression of cytokines including IL-23 and the proform of IL-1β, and IL-23 is an indispensable upstream regulator of IL-17A production in γδT cells.^32^ DAMPs released from injured myocardium could also drive the formation of inflammasomes in cardiomyocytes, fibroblasts, and macrophages, leading to the processing of caspase-1 into its enzymatically active form. This active caspase-1 cleaves pro-IL-1β to release active IL-1β, which can then work in concert with IL-23 to drive IL-17A production in γδT cells.^14,15,34,38^ The γδT cells themselves can directly respond to TLR stimulation in synergy with IL-23. Consistent with our findings, previous studies have revealed that TLR2 or TLR4 deficiency and antibodies targeting IL-1 signaling alleviated cardiac remodeling.^39–42^ Notably, treatment of rats even 24 hours after MI with an IL-1 receptor blocker alleviated LV remodeling without affecting early infarct size.^39^ These findings suggested that the cardioprotective effect of an IL-1 receptor blocker is at least in part mediated by inhibiting the IL-23/IL-17 axis in post-MI heart. In terms of cell migration, FTY720-sensitive S1P receptor signaling helps to mediate γδT cell egress from lymph nodes,^30,43^ whereas CCR6-expressing γδT cells are recruited to the sites of injury that express CCL20 (Figure 15).

Notably, resident cardiac fibroblasts were the major target of IL-17A in stimulating the expression of CXCL1 and CCL2, and cardiac neutrophils constitutively expressed the CXCL1 receptor CXCR2 (Figure 13F). IL-17A is known to promote the expression of CXCL1 by inducing gene transcription and posttranscriptional stabilization of mRNA in fibroblasts.^44,45^ Neutrophils accumulating at injured sites might therefore release proteolytic enzymes or reactive oxygen species to damage surrounding myocytes, which play a crucial role in neutrophil-mediated cardiac injury.^11,46^ Furthermore, IL-17A enhances M1 macrophage polarization and directly activates macrophages to express TNF-α, IL-6, IL-1β, and MMP-9, further exacerbating myocardial damage.^47–50^

Early scar formation at the site of the infarct is important to prevent cardiac rupture, whereas subsequent interstitial fibrosis of adjacent myocardium and increased MMP production associated with a sustained inflammatory response contributes to adverse remodeling later on.^51^ We found that IL-17A plays a crucial role in the expression of MMPs and fibrosis-related genes in infarcted heart only in the late stage of myocardial injury, not earlier on. Consistent with this, we did not see an increased prevalence of cardiac death from cardiac rupture in mice lacking IL-23, IL-17A, or TCRγδ, despite our relatively small sample sizes. In addition, survival benefit in mice lacking IL-23, IL-17A, or TCRγδ became apparent 7 days after MI.

We could not detect γδT cells in the post-MI tissues of patients because no antibody against human TCRγδ was available. However, we localized some IL-17A-expressing T cells in the border and infarct areas of heart sampled from patients after MI (data not shown). Thus, it seems highly likely that infiltrating IL-17A-producing T lymphocytes have a substantial role in post-MI cardiac remodeling in patients.

IL-17A had no effect on the early post-MI inflammatory process, which is part of the healing process. Instead, it seemed to act at a later stage (≍7 days) as effector cytokine-inducing cardiac remodeling and leading to end-stage dilated cardiomyopathy. This finding supports a recent study showing an essential such role for IL-17A in remodeling, but not in the development of autoimmune myocarditis.^19^ This study, together with ours, indicated that the late remodeling–specific effect of IL-17A is a potential therapeutic target for preventing the development of chronic heart failure from postmyocarditis and post-MI cardiac remodeling. To translate these interesting but preliminary findings into the clinic, further human studies with larger sample sizes are needed.

## References

[1] Roger VL Go AS Lloyd-Jones DM Benjamin EJ Berry JD Borden WB Bravata DM Dai S Ford ES Fox CS Fullerton HJ Gillespie C Hailpern SM Heit JA Howard VJ Kissela BM Kittner SJ Lackland DT Lichtman JH Lisabeth LD Makuc DM Marcus GM Marelli A Matchar DB Moy CS Mozaffarian D Mussolino ME Nichol G Paynter NP Soliman EZ Sorlie PD Sotoodehnia N Turan TN Virani SS Wong ND Woo D Turner MB Heart disease and stroke statistics–2012 update: a report from the American Heart Association. Circulation. 2012;125:e2-e220. 22179539 10.1161/CIR.0b013e31823ac046 PMC4440543

[2] Opie LH Commerford PJ Gersh BJ Pfeffer MA Controversies in ventricular remodelling. Lancet. 2006;367:356-367. 16443044 10.1016/S0140-6736(06)68074-4

[3] Krum H Teerlink JR Medical therapy for chronic heart failure. Lancet. 2011;378:713-721. 21856485 10.1016/S0140-6736(11)61038-6

[4] Mudd JO Kass DA Tackling heart failure in the twenty-first century. Nature. 2008;451:919-928. 18288181 10.1038/nature06798

[5] Brown NJ Vaughan DE Angiotensin-converting enzyme inhibitors. Circulation. 1998;97:1411-1420. 9577953 10.1161/01.cir.97.14.1411

[6] Velagaleti RS Pencina MJ Murabito JM Wang TJ Parikh NI D'Agostino RB Levy D Kannel WB Vasan RS Long-term trends in the incidence of heart failure after myocardial infarction. Circulation. 2008;118:2057-2062. 18955667 10.1161/CIRCULATIONAHA.108.784215 PMC2729712

[7] Kleinbongard P Heusch G Schulz R TNFalpha in atherosclerosis, myocardial ischemia/reperfusion and heart failure. Pharmacol Ther. 2010;127:295-314. 20621692 10.1016/j.pharmthera.2010.05.002

[8] Tang TT Yuan J Zhu ZF Zhang WC Xiao H Xia N Yan XX Nie SF Liu J Zhou SF Li JJ Yao R Liao MY Tu X Liao YH Cheng X Regulatory T cells ameliorate cardiac remodeling after myocardial infarction. Basic Res Cardiol. 2012;107:232 22189560 10.1007/s00395-011-0232-6

[9] Arslan F de Kleijn DP Pasterkamp G Innate immune signaling in cardiac ischemia. Nat Rev Cardiol. 2011;8:292-300. 21448140 10.1038/nrcardio.2011.38

[10] Nahrendorf M Pittet MJ Swirski FK Monocytes: protagonists of infarct inflammation and repair after myocardial infarction. Circulation. 2010;121:2437-2445. 20530020 10.1161/CIRCULATIONAHA.109.916346 PMC2892474

[11] Frangogiannis NG The immune system and cardiac repair. Pharmacol Res. 2008;58:88-111. 18620057 10.1016/j.phrs.2008.06.007 PMC2642482

[12] Giugliano GR Giugliano RP Gibson CM Kuntz RE Meta-analysis of corticosteroid treatment in acute myocardial infarction. Am J Cardiol. 2003;91:1055-1059. 12714146 10.1016/s0002-9149(03)00148-6

[13] Iwakura Y Ishigame H Saijo S Nakae S Functional specialization of interleukin-17 family members. Immunity. 2011;34:149-162. 21349428 10.1016/j.immuni.2011.02.012

[14] Sutton CE Lalor SJ Sweeney CM Brereton CF Lavelle EC Mills KH Interleukin-1 and IL-23 induce innate IL-17 production from gammadelta T cells, amplifying Th17 responses and autoimmunity. Immunity. 2009;31:331-341. 19682929 10.1016/j.immuni.2009.08.001

[15] Cai Y Shen X Ding C Qi C Li K Li X Jala VR Zhang HG Wang T Zheng J Yan J Pivotal role of dermal IL-17-producing gammadelta T cells in skin inflammation. Immunity. 2011;35:596-610. 21982596 10.1016/j.immuni.2011.08.001 PMC3205267

[16] Erbel C Dengler TJ Wangler S Lasitschka F Bea F Wambsganss N Hakimi M Bockler D Katus HA Gleissner CA Expression of IL-17A in human atherosclerotic lesions is associated with increased inflammation and plaque vulnerability. Basic Res Cardiol. 2011;106:125-134. 21116822 10.1007/s00395-010-0135-y

[17] Lajoie S Lewkowich IP Suzuki Y Clark JR Sproles AA Dienger K Budelsky AL Wills-Karp M Complement-mediated regulation of the IL-17A axis is a central genetic determinant of the severity of experimental allergic asthma. Nat Immunol. 2010;11:928-935. 20802484 10.1038/ni.1926 PMC2943538

[18] Stromnes IM Cerretti LM Liggitt D Harris RA Goverman JM Differential regulation of central nervous system autoimmunity by T(H)1 and T(H)17 cells. Nat Med. 2008;14:337-342. 18278054 10.1038/nm1715 PMC2813727

[19] Baldeviano GC Barin JG Talor MV Srinivasan S Bedja D Zheng D Gabrielson K Iwakura Y Rose NR Cihakova D Interleukin-17A is dispensable for myocarditis but essential for the progression to dilated cardiomyopathy. Circ Res. 2010;106:1646-1655. 20378858 10.1161/CIRCRESAHA.109.213157

[20] Cua DJ Sherlock J Chen Y Murphy CA Joyce B Seymour B Lucian L To W Kwan S Churakova T Zurawski S Wiekowski M Lira SA Gorman D Kastelein RA Sedgwick JD Interleukin-23 rather than interleukin-12 is the critical cytokine for autoimmune inflammation of the brain. Nature. 2003;421:744-748. 12610626 10.1038/nature01355

[21] Chen Y Wood KJ Interleukin-23 and Th17 cells in transplantation immunity: does 23+17 equal rejection?. Transplantation. 2007;84:1071-1074. 17998858 10.1097/01.tp.0000287126.12083.48

[22] Liao YH Xia N Zhou SF Tang TT Yan XX Lv BJ Nie SF Wang J Iwakura Y Xiao H Yuan J Jevallee H Wei F Shi GP Cheng X Interleukin-17A contributes to myocardial ischemia/reperfusion injury by regulating cardiomyocyte apoptosis and neutrophil infiltration. J Am Coll Cardiol. 2012;59:420-429. 22261166 10.1016/j.jacc.2011.10.863 PMC3262985

[23] Barry SP Ounzain S McCormick J Scarabelli TM Chen-Scarabelli C Saravolatz LI Faggian G Mazzucco A Suzuki H Thiemermann C Knight RA Latchman DS Stephanou A Enhanced IL-17 signalling following myocardial ischaemia/reperfusion injury. Int J Cardiol In press 10.1016/j.ijcard.2011.08.849 PMC3581775 22030025

[24] Nakae S Komiyama Y Nambu A Sudo K Iwase M Homma I Sekikawa K Asano M Iwakura Y Antigen-specific T cell sensitization is impaired in IL-17-deficient mice, causing suppression of allergic cellular and humoral responses. Immunity. 2002;17:375-387. 12354389 10.1016/s1074-7613(02)00391-6

[25] Shichita T Hasegawa E Kimura A Morita R Sakaguchi R Takada I Sekiya T Ooboshi H Kitazono T Yanagawa T Ishii T Takahashi H Mori S Nishibori M Kuroda K Akira S Miyake K Yoshimura A Peroxiredoxin family proteins are key initiators of post-ischemic inflammation in the brain. Nat Med. 2012;18:911-917. 22610280 10.1038/nm.2749

[26] Anzai A Anzai T Nagai S Maekawa Y Naito K Kaneko H Sugano Y Takahashi T Abe H Mochizuki S Sano M Yoshikawa T Okada Y Koyasu S Ogawa S Fukuda K Regulatory role of dendritic cells in postinfarction healing and left ventricular remodeling. Circulation. 2012;125:1234-1245. 22308302 10.1161/CIRCULATIONAHA.111.052126

[27] Shichita T Sugiyama Y Ooboshi H Sugimori H Nakagawa R Takada I Iwaki T Okada Y Iida M Cua DJ Iwakura Y Yoshimura A Pivotal role of cerebral interleukin-17-producing gammadeltat cells in the delayed phase of ischemic brain injury. Nat Med. 2009;15:946-950. 19648929 10.1038/nm.1999

[28] Ieda M Tsuchihashi T Ivey KN Ross RS Hong TT Shaw RM Srivastava D Cardiac fibroblasts regulate myocardial proliferation through beta1 integrin signaling. Dev Cell. 2009;16:233-244. 19217425 10.1016/j.devcel.2008.12.007 PMC2664087

[29] Tokudome S Sano M Shinmura K Matsuhashi T Morizane S Moriyama H Tamaki K Hayashida K Nakanishi H Yoshikawa N Shimizu N Endo J Katayama T Murata M Yuasa S Kaneda R Tomita K Eguchi N Urade Y Asano K Utsunomiya Y Suzuki T Taguchi R Tanaka H Fukuda K Glucocorticoid protects rodent hearts from ischemia/reperfusion injury by activating lipocalin-type prostaglandin D synthase-derived PGD2 biosynthesis. J Clin Invest. 2009;119:1477-1488. 19451694 10.1172/JCI37413 PMC2689117

[30] Mandala S Hajdu R Bergstrom J Quackenbush E Xie J Milligan J Thornton R Shei GJ Card D Keohane C Rosenbach M Hale J Lynch CL Rupprecht K Parsons W Rosen H Alteration of lymphocyte trafficking by sphingosine-1-phosphate receptor agonists. Science. 2002;296:346-349. 11923495 10.1126/science.1070238

[31] Esplugues E Huber S Gagliani N Hauser AE Town T Wan YY O'Connor W Jr Rongvaux A Van Rooijen N Haberman AM Iwakura Y Kuchroo VK Kolls JK Bluestone JA Herold KC Flavell RA Control of Th17 cells occurs in the small intestine. Nature. 2011;475:514-518. 21765430 10.1038/nature10228 PMC3148838

[32] Rubino SJ Geddes K Girardin SE Innate IL-17 and IL-22 responses to enteric bacterial pathogens. Trends Immunol. 2012;33:112-118. 22342740 10.1016/j.it.2012.01.003

[33] Ahrens S Zelenay S Sancho D Hanc P Kjaer S Feest C Fletcher G Durkin C Postigo A Skehel M Batista F Thompson B Way M Sousa CRE Schulz O F-actin is an evolutionarily conserved damage-associated molecular patternrecognized by DNGR-1, a receptor for dead cells. Immunity. 2012;36:635-645. 22483800 10.1016/j.immuni.2012.03.008

[34] Mezzaroma E Toldo S Farkas D Seropian IM Van Tassell BW Salloum FN Kannan HR Menna AC Voelkel NF Abbate A The inflammasome promotes adverse cardiac remodeling following acute myocardial infarction in the mouse. Proc Natl Acad Sci USA. 2011;108:19725-19730. 22106299 10.1073/pnas.1108586108 PMC3241791

[35] Oka T Hikoso S Yamaguchi O Taneike M Takeda T Tamai T Oyabu J Murakawa T Nakayama H Nishida K Akira S Yamamoto A Komuro I Otsu K Mitochondrial DNA that escapes from autophagy causes inflammation and heart failure. Nature. 2012;485:251-255. 22535248 10.1038/nature10992 PMC3378041

[36] Piccinini AM Midwood KS DAMPening inflammation by modulating TLR signalling. Mediators Inflamm. 2010 1-21 doi:10.1155/2010/672395 10.1155/2010/672395 PMC2913853 20706656

[37] Volz HC Laohachewin D Seidel C Lasitschka F Keilbach K Wienbrandt AR Andrassy J Bierhaus A Kaya Z Katus HA Andrassy M S100A8/A9 aggravates post-ischemic heart failure through activation of RAGE-dependent NF-kappab signaling. Basic Res Cardiol. 2012;107:250 22318783 10.1007/s00395-012-0250-z

[38] Strowig T Henao-Mejia J Elinav E Flavell R Inflammasomes in health and disease. Nature. 2012;481:278-286. 22258606 10.1038/nature10759

[39] Abbate A Salloum FN Vecile E Das A Hoke NN Straino S Biondi-Zoccai GG Houser JE Qureshi IZ Ownby ED Gustini E Biasucci LM Severino A Capogrossi MC Vetrovec GW Crea F Baldi A Kukreja RC Dobrina A Anakinra, a recombinant human interleukin-1 receptor antagonist, inhibits apoptosis in experimental acute myocardial infarction. Circulation. 2008;117:2670-2683. 18474815 10.1161/CIRCULATIONAHA.107.740233

[40] Abbate A Van Tassell BW Seropian IM Toldo S Robati R Varma A Salloum FN Smithson L Dinarello CA Interleukin-1beta modulation using a genetically engineered antibody prevents adverse cardiac remodelling following acute myocardial infarction in the mouse. Eur J Heart Fail. 2010;12:319-322. 20335350 10.1093/eurjhf/hfq017

[41] Timmers L Sluijter JP van Keulen JK Hoefer IE Nederhoff MG Goumans MJ Doevendans PA van Echteld CJ Joles JA Quax PH Piek JJ Pasterkamp G de Kleijn DP Toll-like receptor 4 mediates maladaptive left ventricular remodeling and impairs cardiac function after myocardial infarction. Circ Res. 2008;102:257-264. 18007026 10.1161/CIRCRESAHA.107.158220

[42] Shishido T Nozaki N Yamaguchi S Shibata Y Nitobe J Miyamoto T Takahashi H Arimoto T Maeda K Yamakawa M Takeuchi O Akira S Takeishi Y Kubota I Toll-like receptor-2 modulates ventricular remodeling after myocardial infarction. Circulation. 2003;108:2905-2910. 14656915 10.1161/01.CIR.0000101921.93016.1C

[43] Theilmeier G Schmidt C Herrmann J Keul P Schafers M Herrgott I Mersmann J Larmann J Hermann S Stypmann J Schober O Hildebrand R Schulz R Heusch G Haude M von Wnuck Lipinski K Herzog C Schmitz M Erbel R Chun J Levkau B High-density lipoproteins and their constituent, sphingosine-1-phosphate, directly protect the heart against ischemia/reperfusion injury in vivo via the S1P3 lysophospholipid receptor. Circulation. 2006;114:1403-1409. 16982942 10.1161/CIRCULATIONAHA.105.607135

[44] Datta S Novotny M Pavicic PG Jr Zhao C Herjan T Hartupee J Hamilton T IL-17 regulates CXCL1 mRNA stability via an AUUUA/tristetraprolin-independent sequence. J Immunol. 2010;184:1484-1491. 20042592 10.4049/jimmunol.0902423 PMC2829999

[45] Sun D Novotny M Bulek K Liu C Li X Hamilton T Treatment with IL-17 prolongs the half-life of chemokine CXCL1 mRNA via the adaptor TRAF5 and the splicing-regulatory factor SF2 (ASF). Nat Immunol. 2011;12:853-860. 21822258 10.1038/ni.2081 PMC3597344

[46] Vinten-Johansen J Involvement of neutrophils in the pathogenesis of lethal myocardial reperfusion injury. Cardiovasc Res. 2004;61:481-497. 14962479 10.1016/j.cardiores.2003.10.011

[47] Marchant DJ Boyd JH Lin DC Granville DJ Garmaroudi FS McManus BM Inflammation in myocardial diseases. Circ Res. 2012;110:126-144. 22223210 10.1161/CIRCRESAHA.111.243170

[48] Coggins M Rosenzweig A The fire within: cardiac inflammatory signaling in health and disease. Circ Res. 2012;110:116-125. 22223209 10.1161/CIRCRESAHA.111.243196

[49] Hu Y Zhang H Lu Y Bai H Xu Y Zhu X Zhou R Ben J Chen Q Class a scavenger receptor attenuates myocardial infarction-induced cardiomyocyte necrosis through suppressing M1 macrophage subset polarization. Basic Res Cardiol. 2011;106:1311-1328. 21769674 10.1007/s00395-011-0204-x

[50] Dayan V Yannarelli G Billia F Filomeno P Wang XH Davies JE Keating A Mesenchymal stromal cells mediate a switch to alternatively activated monocytes/macrophages after acute myocardial infarction. Basic Res Cardiol. 2011;106:1299-1310. 21901289 10.1007/s00395-011-0221-9

[51] Frangogiannis NG Regulation of the inflammatory response in cardiac repair. Circ Res. 2012;110:159-173. 22223212 10.1161/CIRCRESAHA.111.243162 PMC3690135

